# Gelled Oil-in-Water Microemulsions Stabilized with Natural Surfactant Decyl Glucoside as Cutaneous Nanocarriers for Cinnamon Bark Essential Oil: Formulation and Comprehensive Pharmacotechnical Characterization

**DOI:** 10.3390/pharmaceutics18101317

**Published:** 2026-10-17

**Authors:** Milja Živković, Georgeta-Hermina Coneac, Ana Barjaktarević, Snežana Cupara, Ana-Maria Cotan, Lavinia-Lia Vlaia, Monica Elisabeta Maxim, Aurica Precupas, Ksenija Obradović, Marina Kostić, Vicenţiu Vlaia, Ştefania Neamu, Ioana-Viorica Olariu

**Affiliations:** 1 Department of Pharmacy, Faculty of Medical Sciences, University of Kragujevac, 34000 Kragujevac, Serbia; zivkovicmilja@gmail.com (M.Ž.); cupara@fmn.kg.ac.rs (S.C.); ksenija.kojicic@gmail.com (K.O.); 2 Department II—Pharmaceutical Technology, Formulation and Technology of Drugs Research Center, “Victor Babes” University of Medicine and Pharmacy, Eftimie Murgu Square No. 2, 300041 Timisoara, Romania; coneac.georgeta@umft.ro (G.-H.C.); mut.anamaria@umft.ro (A.-M.C.); vlaia.lavinia@umft.ro (L.-L.V.); olariu.ioana@umft.ro (I.-V.O.); 3 Center for Molecular Medicine and Stem Cell Research, Faculty of Medical Sciences, University of Kragujevac, 34000 Kragujevac, Serbia; 4 Laboratory of Colloid Chemistry, “Ilie Murgulescu” Institute of Physical Chemistry, Romanian Academy, Splaiul Independentei 202, 060021 Bucharest, Romania; monimaxim@gmail.com; 5 “Ilie Murgulescu” Institute of Physical Chemistry, Romanian Academy, Splaiul Independentei 202, 060021 Bucharest, Romania; aprecupas@icf.ro; 6 Department of Pharmacology and Toxicology, Faculty of Medical Sciences, University of Kragujevac, 34000 Kragujevac, Serbia; marrina2006kg@yahoo.com; 7 Department II—Organic Chemistry, Formulation and Technology of Drugs Research Center, “Victor Babes” University of Medicine and Pharmacy, Eftimie Murgu Square No. 2, 300041 Timisoara, Romania; 8 Pharmacy Doctoral School, Formulation and Technology of Drugs Research Center, Faculty of Pharmacy, “Victor Babes” University of Medicine and Pharmacy, 300041 Timisoara, Romania; adriana.colgiu@umft.ro

**Keywords:** cinnamon essential oil, decyl glucoside, gelled microemulsion, oil-in-water microemulsion, cutaneous delivery, skin permeation

## Abstract

**Background/Objectives**: Cinnamon bark essential oil (CEO) possesses antimicrobial, antioxidant, and anti-inflammatory properties. However, its topical application is limited by poor aqueous solubility, volatility, and chemical instability. The aim of this study was to develop gelled oil-in-water microemulsions stabilized with Decyl Glucoside for the cutaneous delivery of CEO and to evaluate their physicochemical and pharmaceutical characteristics. **Methods**: Pseudoternary phase diagrams were constructed to identify the microemulsion regions using Decyl Glucoside, propylene glycol, CEO, isopropyl myristate, and water. Selected CEO-loaded microemulsions were characterized in terms of droplet size, polydispersity index, zeta potential, refractive index, viscosity, and apparent pH, and were subsequently gelled with Aerosil^®^ 200. The resulting gelled microemulsions were evaluated for CEO content, apparent pH, rheological and textural properties, spreadability, in vitro release and skin permeation studies. The short-term stability of both liquid and gelled microemulsions was also assessed over 45 days. **Results**: Three CEO-loaded oil-in-water microemulsions were selected from the isotropic regions of the pseudoternary phase diagrams and successfully transformed into homogeneous gelled systems without phase separation. CEO incorporation increased the storage modulus and yield stress of the gelled formulations compared with the corresponding blank systems. The surfactant-to-cosurfactant ratio markedly influenced the rheological properties, mechanical strength, and in vitro release and skin permeation of CEO. Among the investigated formulations, MEG-CEO-2 provided the best balance between mechanical properties, CEO release, and skin permeation. Both liquid and gelled microemulsions maintained their principal physicochemical characteristics during the 45-day stability assessment. **Conclusions**: Decyl Glucoside-stabilized gelled microemulsions exhibited physicochemical and pharmaceutical properties supporting their further investigation as topical delivery systems for CEO. The results demonstrate that the surfactant-to-cosurfactant ratio can be used to tailor formulation performance, supporting a rational approach for the development of CEO-loaded dermal delivery systems. However, preservation of the biological activities of CEO following incorporation into these systems remains to be experimentally established.

## 1. Introduction

In recent years, interest in plant-derived bioactive compounds for dermatological and cosmetic formulations has grown, primarily because of their antioxidant, antimicrobial, and anti-inflammatory properties. Among these, cinnamon (*Cinnamomum verum* J.Presl., syn. *Cinnamomum zeylanicum* Blume) bark essential oil has attracted considerable attention owing to its rich phytochemical composition, characterized by a high content of cinnamaldehyde and various phenolic constituents, collectively contributing to its wide spectrum of biological activities [1,2]. Besides their biological activity, the successful development of topical formulations containing essential oils requires careful consideration of formulation parameters that influence both product quality and skin compatibility. Among these, pH represents an important quality attribute, as topical formulations are generally recommended to possess a pH close to that of the physiological skin surface to preserve skin barrier function and minimize irritation [3,4]. Despite their promising biological profile, the incorporation of essential oils into topical formulations remains challenging because their volatility, susceptibility to oxidation, and limited aqueous solubility may compromise formulation stability and dermal delivery. These limitations necessitate the development of suitable delivery systems capable of improving stability, modulating release, and enhancing dermal penetration of active compounds [5,6]. Microemulsions have emerged as particularly attractive carriers for lipophilic bioactive compounds due to their thermodynamic stability, optical transparency, low interfacial tension, and ability to form nanosized droplets spontaneously. These unique characteristics provide high solubilization capacity for poorly water-soluble compounds while facilitating close contact between the incorporated active ingredients and the skin surface. Lawrence and Rees emphasized that the bicontinuous or oil-in-water structure of microemulsions enables efficient solubilization of both hydrophilic and lipophilic molecules. In contrast, their dynamic internal structure promotes enhanced diffusion and transport across biological membranes, making them highly promising delivery systems for dermal and transdermal applications [7]. The suitability of microemulsions as carriers for cinnamon bark essential oil (CEO) has also been demonstrated. For example, CEO has been successfully incorporated into microemulsion systems exhibiting droplet sizes below 60 nm, favorable physicochemical characteristics, enhanced tyrosinase inhibitory activity, and reduced cytotoxicity compared with the free essential oil, highlighting the potential of microemulsification to improve the functionality of this natural bioactive ingredient [8]. Consequently, microemulsions can improve drug partitioning into the stratum corneum, enhance skin permeation, and increase the bioavailability of incorporated compounds. Their suitability for the delivery of essential oils has also been demonstrated in recent studies. In particular, Vlaia et al. developed biocompatible chitosan-gelled microemulsions based on essential oils and sucrose ester surfactants, demonstrating favorable physicochemical stability, appropriate rheological properties, and sustained release characteristics, highlighting their potential as efficient nanocarriers for topical drug delivery. To further optimize their applicability for dermal use, microemulsions can be transformed into gel microemulsions by incorporating suitable gelling agents. This approach combines the advantages of microemulsions with improved rheological behavior, enhanced retention at the site of application, and better patient compliance [9]. The present study explored the feasibility of using decyl glucoside as the sole surfactant for the development of CEO-loaded cutaneous microemulsions. Decyl glucoside belongs to the family of alkyl polyglucosides (APGs), a class of nonionic surfactants that has attracted considerable attention in recent years because of its favorable toxicological and environmental profile [10]. Owing to their renewable origin, excellent biodegradability, low toxicity, and high skin compatibility, APGs are increasingly employed in cosmetic, personal care, and pharmaceutical formulations [11]. Furthermore, according to the Cosmetic Ingredient Review (CIR) Expert Panel, alkyl polyglucosides exhibit a favorable safety profile for topical use. They are considered non-irritating under the recommended conditions of use [12]. Despite the growing interest in APG-based microemulsions, their use as dermal delivery systems remains comparatively limited. Previous studies have demonstrated the potential of APG-containing microemulsions for topical delivery of chemically diverse cargos, including ascorbic acid, tacrolimus, lycopene, adapalene, and other poorly water-soluble drugs [10,13,14,15,16]. APG-based microemulsions containing tea tree essential oil have also been investigated for potential personal-care applications [17]. However, these studies have predominantly focused either on liquid microemulsion systems or on the delivery of individual active compounds. Cinnamon bark essential oil represents a more complex formulation challenge because it is a multicomponent, highly lipophilic, volatile, and chemically labile cargo, dominated by cinnamaldehyde. Its incorporation therefore requires not only efficient solubilization but also control of interfacial organization, formulation structure, release behavior, and cutaneous transport. Accordingly, the present study was designed not merely to demonstrate the feasibility of incorporating CEO into an APG-based microemulsion, but to investigate how the decyl glucoside-to-propylene glycol ratio governs a broader composition-structure-performance relationship. The selected microemulsions were subsequently converted into Aerosil^®^ 200-gelled semisolid systems and comprehensively evaluated in terms of physicochemical properties, rheological and mechanical behavior, in vitro release, and ex vivo skin permeation. This integrated approach was intended to provide a rational framework for tuning APG-based dermal delivery systems rather than simply developing another microemulsion formulation.

## 2. Materials and Methods

### 2.1. Materials

Cinnamon bark essential oil (CEO) was purchased from Fares Bio Vital Laboratories (Orăştie, Romania). According to the manufacturer’s certificate of analysis for the batch used in this study [18], cinnamaldehyde was the predominant constituent (75.911%), together with linalool (5.505%), and eugenol (4.297%). The certificate also specified a solubility of 1:2 (*v*/*v*) in 70% (*v*/*v*) ethanol. The analyzed batch complied with the manufacturer’s specifications. Isopropyl myristate (IPM) was kindly donated by Cognis (Monheim, Germany) and natural surfactant decyl glucoside (Decyl Surfactant) was provided by Ellemental (Oradea, Romania). Propylene glycol (PG) was purchased from Sigma-Aldrich Chemie GmbH (Steinheim, Germany), diethyleneglycol monoethyl ether (DEGEE) from Acros Organics (Geel, Belgium), ethanol from Girelli Alcool SRL (San Giacomo, Italy) and fumed silica (Aerosil^®^ 200 Pharma) was received as a gift sample from Evonik Industries AG (Hanau, Germany). Distilled water was used to prepare the microemulsions and gelled microemulsions, and bidistilled water was used to prepare the 0.9% isotonic sodium chloride solution. Polyethersulfone hydrophilic synthetic membranes (Supor membranes, 0.45 µm in pore size and 25 mm in diameter) were supplied by Pall Corporation (Port Washington, NY, USA), and the porcine skin was excised from pork ears purchased from a local slaughterhouse. Except for the porcine skin, all the materials used in the study were of pharmaceutical or analytical purity and were used as received.

### 2.2. Methods

#### 2.2.1. Construction of Pseudo-Ternary Phase Diagrams

To define the microemulsion region and the compositional ranges of the oil phase, aqueous phase, and surfactant/cosurfactant mixture (S_mix_), pseudo-ternary phase diagrams were constructed. The oil phase consisted of CEO and isopropyl myristate (2:1, *w*/*w*); decyl glucoside (DG) was used as the surfactant, and propylene glycol (PG) or diethylene glycol monoethyl ether (DEGEE) as cosurfactant. DG was mixed with each cosurfactant at mass ratios of 1:1, 1.5:1 and 2:1. Since the commercial Decyl Surfactant contains 53.4% decyl glucoside and 46.6% water, the S_mix_ ratios were corrected to account for the pure decyl glucoside, as previously reported [14,19]. Oil phase/S_mix_ blends were prepared at nine different weight ratios (1:9, 2:8, 3:7, 4:6, 5:5, 6:4, 7:3, 8:2 and 9:1), covering a broad composition range for accurate mapping of the pseudo-ternary phase diagram.

Pseudo-ternary phase diagrams were constructed using a miniaturized water titration method adapted from the microplate dilution approach reported by Schmidts et al. [19]. Predetermined oil phase/S_mix_ blends were transferred into a 96-well microplate (350 µL/well), followed by stepwise addition of water to obtain a wide range of oil/S_mix_/water compositions. The microplates were sealed and equilibrated at 25 ± 0.5 °C in a thermostatically controlled shaker, and the samples were visually evaluated for transparency, optical homogeneity, fluidity and phase separation. Clear, isotropic single-phase mixtures were classified as microemulsions and used to construct the pseudo-ternary phase diagrams, whereas turbid or phase-separated samples were excluded.

The areas of the microemulsion regions were calculated according to the coordinate-geometry approach proposed by Berkman and Güleç [20]. Briefly, the experimentally determined points delimiting each microemulsion region were converted from ternary composition data into Cartesian x- and y-coordinates. The coordinates of the vertices defining the resulting closed polygon corresponding to the microemulsion region were subsequently used to calculate its area using Gauss’s area formula (Shoelace formula) in Microsoft Excel. As the pseudoternary diagram was represented as an equilateral triangle with a side length of 100 arbitrary coordinate units, the calculated microemulsion areas were expressed as squared coordinate units (units^2^).

#### 2.2.2. Preparation of O/W Cinnamon Bark Essential Oil-Loaded Microemulsions

Only the pseudo-ternary phase diagrams corresponding to the DG/PG systems were used for ME formulation selection. One ME formulation from each pseudo-ternary phase diagram was selected to evaluate the effect of the surfactant/cosurfactant ratio. All selected formulations contained identical proportions of the oil phase (4.5% *w*/*w*) and S_mix_ (45% *w*/*w*), differing only in the DG:PG weight ratio within S_mix_ (1:1, 1.5:1, and 2:1). Thus, differences in the physicochemical, rheological, textural, and release/permeation properties could be attributed to variations in the surfactant/cosurfactant ratio. The composition of the experimental MEs loaded with CEO is shown in Table 1.

Three microemulsion formulations were prepared by mixing the cinnamon essential oil-isopropyl myristate oil phase with the corresponding decyl glucoside/propylene glycol mixture (S_mix_). The resulting binary mixture was gradually diluted with purified water, which was incorporated dropwise under continuous magnetic stirring (400 rpm) at room temperature (25 ± 1 °C) using a magnetic stirrer (MR Hei-Tec, Heidolph Instruments GmbH&Co.KG, Schwabach, Germany), until clear and optically homogeneous systems were obtained. The CEO concentration was fixed at 3% (*w*/*w*) in all formulations. The freshly prepared microemulsions were filled into amber glass containers and allowed to equilibrate for 24 h at room temperature prior to physicochemical, rheological, textural and in vitro CEO release/permeation characterization.

#### 2.2.3. Preparation of Gelled Cinnamon Bark Essential Oil-Loaded Microemulsions

The CEO-loaded microemulsions were converted into gelled systems by gradually dispersing Aerosil^®^ 200 (hydrophilic fumed silica) into the formulations under continuous magnetic stirring (400 rpm) at room temperature. Stirring was continued for approximately 1 h, until homogeneous gelled microemulsions were obtained, after which the formulations were allowed to equilibrate for 48 h before further characterization. The final concentration of Aerosil^®^ 200 in all formulations was 4.0% (*w*/*w*).

#### 2.2.4. Physicochemical Characterization of O/W Cinnamon Bark Essential Oil-Loaded Microemulsions

The organoleptic properties of the ME formulations were evaluated by visual and olfactory inspection, considering their appearance, color, odor, and flow behavior.

*Droplet size, polydispersity index, and zeta potential determination*

The mean droplet diameter, polydispersity index (PDI), and zeta potential of the CEO-loaded microemulsions were determined by dynamic light scattering (DLS) and electrophoretic light scattering (ELS), using a Zetasizer Nano ZS instrument (Malvern Instruments Ltd., Malvern, UK). Measurements were performed in triplicate at 25 °C using a fixed detection angle of 173 º, and the results were expressed as mean ± standard deviation.

*Refractive index determination*

The refractive index of the CEO-loaded microemulsions was determined using a digital refractometer (Reichert ABBE Mark II, Depew, NY, USA). All measurements were performed in triplicate at 25 °C.

*Viscosity measurements*

The viscosity of the CEO-loaded ME formulations was determined using a Brookfield rotational viscosimeter (DV-I+, Brookfield Engineering Laboratories, Middleboro, MA, USA), equipped with a S-05 spindle. Measurements were carried out in triplicate at 25 ± 2 °C.

*Apparent pH measurements*

The apparent pH of the CEO-loaded microemulsion was potentiometrically determined at 25 °C, using a calibrated SevenExcellence^TM^ S400-KIT pH meter (Mettler Toledo, Columbus, OH, USA). Each sample was analyzed in triplicate.

#### 2.2.5. Characterization of Gelled Cinnamon Bark Essential Oil-Loaded Microemulsions

*Determination of cinnamon bark essential oil content*

The CEO content of the gelled CEO-loaded microemulsions was determined by UV-VIS spectrophotometry. Briefly, 0.3 g of each formulation was accurately weighed into a 25 mL volumetric flask and diluted to volume with 95% (*v*/*v*) ethanol, filtered through a 0.45 μm membrane filter, and analyzed at 292 nm using a UV-VIS spectrophotometer (T70+, PG Instruments Ltd., Leicestershire, UK). Quantification was performed using a calibration curve prepared in 95% (*v*/*v*) ethanol (R^2^ = 0.9992). All determinations were performed in triplicate.

*Apparent pH determination of gelled cinnamon bark essential oil-loaded microemulsions*

The apparent pH of the gelled CEO-loaded MEs and corresponding control formulations was determined using aqueous dispersions containing 5% (*w*/*w*) of each formulation. The dispersions were prepared immediately prior to analysis, and the apparent pH was potentiometrically measured at 25 °C, using a calibrated SevenExcellence^TM^ S400-KIT pH meter (Mettler Toledo, Columbus, OH, USA). All measurements were performed in triplicate.

#### 2.2.6. Rheological Characterization

The rheological properties of the gelled CEO-loaded MEs were investigated using a stress-controlled rheometer (RheoStress 1, Thermo Haake, Karlsruhe, Germany) equipped with a titanium cone-and-plate measuring system (C35/2 º Ti; 35 mm diameter, 2 º cone angle, and 0.106 mm gap). Steady-state shear measurements and dynamic oscillatory tests, including stress and frequency sweep tests, were performed. Corresponding blank gelled microemulsions were analyzed under identical conditions for comparison. All measurements were performed at 23 °C.

*Steady-state shear measurements*

The flow behavior and apparent viscosity of the gelled CEO-loaded MEs were evaluated under controlled shear rate conditions using a continuous ascending-descending shear rate ramp from 0.05 s^−1^ to 100 s^−1^. Flow curves and viscosity profiles were recorded during consecutive ascending and descending shear rate ramps to characterize the flow behavior of the formulations and to evaluate the presence of time-dependent rheological effects. To assess the time-dependent rheological behavior of the formulations, the areas under the ascending (A_UP_) and descending (A_Down_) flow curves were determined, and the relative hysteresis area (A_R_) or thixotropy index was calculated according to Equation (1):

(1) $$A_{R}=\frac{\left(A_{\mathrm{UP}}\;-\;A_{Down}\right)}{A_{\mathrm{UP}}}\cdot 100$$

Formulations were considered thixotropic when A_R_ exceeded 5%, whereas values below 5% were interpreted as indicating pseudoplastic behavior with negligible thixotropy [21].

Rheological data acquisition and data analysis were performed using Haake RheoWin Data Manager software (version 4.3).

*Small-amplitude oscillatory tests*

*Stress sweep test*

The linear viscoelastic region (LVR) of the gelled CEO-loaded microemulsions was determined by oscillatory stress sweep measurements performed at a constant frequency of 1.0 Hz over shear stress ranges of 0.5–10, 0.5–100 and 0.5–500 Pa. The LVR was identified from the stress-independent values of the storage (G’) and loss moduli (G”).

*Frequency sweep test*

Following LVR determination, oscillatory frequency sweep measurements were performed at a constant shear stress of 1 Pa, selected within the linear viscoelastic region, over the frequency range of 0.05–50 Hz. The storage modulus (G’), loss modulus (G”), and complex viscosity (η*) were recorded as functions of oscillation frequency [22].

*Yield stress determination*

The yield stress (τ_0_) was subsequently determined by oscillatory stress sweep measurements at a constant frequency of 1 Hz with applied shear stress increased from 1 to 500 Pa. The yield stress was defined as the stress corresponding to the onset of a pronounced decrease in the storage modulus (G’) [23]. All steady-state and oscillatory rheological experiments were carried out in triplicate.

#### 2.2.7. Consistency Measurements

The consistency-related properties of the gelled CEO-loaded MEs and corresponding blank formulations were characterized using two complementary analytical approaches. The pharmacopeial penetrometric method was used to assess formulation hardness, whereas the parallel-plate technique was applied to evaluate spreadability under progressively increasing compressive loads.

Hardness was determined by penetrometry using a PNR 12 penetrometer (Petrolab, Speyer, Germany) equipped with a standardized microcone and sample container. Measurements were performed at 25 ± 2 °C in accordance with the procedure specified in the European and United States Pharmacopoeias [24,25]. The penetration depth of the cone was recorded in millimeters (mm), and the results were expressed as the mean ± standard deviation (SD) of three independent determinations.

Spreadability of the gelled CEO-loaded MEs was evaluated using the del Pozo Ojeda-Suñé Arbussá extensometer according to a previously described method [26]. Briefly, 1.00 g of each formulation was placed between two parallel plates and subjected to standardized incremental loads. The spread area produced at each applied load was measured and used to construct the spreadability profile. The results were expressed as mean ± SD, and all determinations were performed in triplicate at 25 ± 2 °C.

#### 2.2.8. Texture Analysis

Texture analysis was performed to evaluate the mechanical properties of all gelled microemulsions (CEO-loaded and plain formulations). The textural properties of the gelled CEO-loaded microemulsion formulations were evaluated using a texture analyzer (TA.XT Plus, Stable Micro Systems, London, UK) equipped with a 5 kg load cell. Two complementary tests were performed to evaluate the mechanical behavior of the formulations: a back extrusion compression and a spreadability test.

The back extrusion compression test was carried out using a cylindrical extrusion probe (35 mm diameter) at room temperature. Approximately 50 g of each formulation was carefully transferred into a 100 mL cylindrical container (50 mm sample height), avoiding air entrapment. Following contact detection at a trigger force of 5 g, the probe penetrated the sample to a depth of 15 mm at a constant speed of 2 mm s^−1^ and subsequently returned to its initial position at the same speed. Force-time curves were recorded and analyzed using Texture Exponent software (version 3.0.5.0, Stable Micro Systems). The mechanical parameters obtained from the force-time profiles included firmness (maximum compression force), consistency (positive area under the force-time curve, corresponding to the work required for sample compression), cohesiveness (maximum withdrawal force) and viscosity index (negative area under the force-time curve) [27].

Spreadability was evaluated using a TTC Spreadability Rig (HDP/SR, Stable Micro Systems, London, UK) consisting of complementary male and female conical accessories. The sample was placed into the female cone, and the male cone was moved at a constant speed of 3 mm s^−1^ until complete compression. Force–time profiles were recorded and analyzed using the instrument software (Texture Exponent software, version 3.0.5.0, Stable Micro Systems). Maximum compression force and the positive area under the force–time curve (shear work) were used as spreadability parameters. Lower values of both parameters indicate a lower resistance to deformation and, consequently, improved spreadability of the gelled microemulsions.

For both texture analysis methods, four independent samples of each formulation were analyzed at ambient temperature. The results were expressed as mean ± SD.

#### 2.2.9. Preliminary Short-Term Physicochemical Stability Assessment

The short-term stability of the CEO-loaded microemulsions (ME-CEO-1, ME-CEO-2, and ME-CEO-3) and the corresponding CEO-loaded gelled microemulsions was evaluated over a 45-day storage period. After preparation, the formulations were transferred into tightly closed amber glass containers and stored at 25 °C, protected from light. The same formulation samples were evaluated immediately after preparation (Day 0) and after 15, 30, and 45 days of storage. Day 0 measurements, reported in the corresponding initial characterization sections, were considered baseline values.

The short-term physicochemical stability of the CEO-loaded microemulsions was assessed by monitoring macroscopic appearance, droplet diameter, polydispersity index, dynamic viscosity, zeta potential, and apparent pH. Macroscopic examination included evaluation of transparency, homogeneity, color, and the occurrence of phase separation, sedimentation, or creaming.

The short-term physicochemical stability of the CEO-loaded gelled microemulsions was evaluated by monitoring macroscopic appearance, CEO content, apparent pH, apparent viscosity, and penetration value. Macroscopic examination focused on the maintenance of a homogeneous semisolid appearance and the occurrence of visible phase separation or other macroscopic signs of physical instability. All quantitative parameters were determined using the same experimental procedure and instrumental conditions described in the corresponding characterization sections.

#### 2.2.10. In Vitro Cinnamon Bark Essential Oil Release and Permeation Studies

*Selection of the receptor media for in vitro release and permeation studies*

Before the in vitro release and skin permeation studies, suitable receptor media were selected based on the apparent solubilization capacity of different hydroethanolic media toward CEO to ensure sink conditions. Since the manufacturer reports CEO to be readily soluble in 70% (*v*/*v*) ethanol (1:2, *v*/*v*, according to the certificate of analysis), phosphate-buffered saline (PBS, pH 7.4) and physiological saline containing different ethanol concentrations (30%, 60% or 70% *v*/*v*) were comparatively evaluated as potential receptor media.

The apparent solubility of CEO was determined according to the qualitative solubility approach described in the European Pharmacopoeia [28]. Briefly, 100 µL of CEO was accurately transferred into a glass vial using a calibrated Hamilton microsyringe (725 N, 250 µL, Hamilton Central Europe S.R.L., Giarmata, Romania). The selected hydroethanolic medium was then added incrementally in 5 µL portions under manual shaking after each addition until the initially transparent mixture became persistently opalescent, indicating the apparent solubility limit. All experiments were carried out at room temperature (25 ± 2 °C). The apparent solubility was expressed as the total volume of receptor medium required to reach the onset of persistent opalescence for 100 µL of essential oil. Each determination was performed in triplicate, and the results were expressed as mean ± SD. The receptor medium providing the highest CEO solubilization capacity was selected for subsequent diffusion studies.

Sink conditions were defined relative to the maximum theoretical CEO concentration that could be reached in the receptor compartment, assuming complete release of the CEO initially present in the donor formulation. The maximum theoretical CEO concentration (C_max_) was calculated according to Equation (2):

(2) C_max_ = (m_f_ · w_CEO_)/(ρ_CEO_ · V_R_)

where m_f_ is the maximum mass of gelled microemulsion applied to the donor compartment (0.300 g), w_CEO_ is the mass fraction of CEO in the formulation (0.03), ρ_CEO_ is the density of CEO (1.026 g/mL), and V_R_ is the receptor compartment volume (6.5 mL). Accordingly, C_max_ was 0.00135 mL CEO/mL receptor medium.

The minimum CEO solubility required to satisfy a given sink condition was subsequently calculated as:

(3) S_sink_ = N · C_max_

where S_sink_ is the minimum CEO solubility required in the receptor medium and N is the sink factor (3 or 10). The corresponding solubility thresholds were therefore 0.00405 and 0.0135 mL CEO/mL receptor medium for three-fold and ten-fold sink conditions, respectively. Since the experimental solubility screening was expressed as the volume of receptor medium required to dissolve 0.1 mL CEO, these thresholds corresponded to 24.69 and 7.41 mL of receptor medium, respectively.

*In vitro cinnamon bark essential oil release studies*

The in vitro release of CEO from the gelled microemulsions was investigated using a six-cell vertical Franz diffusion system (Microette-Hanson, model 57-6AS9, Hanson Research, Chatsworth, CA, USA) with an effective diffusion area of 1.762 cm^2^ and receptor volume of 6.5 mL. Hydrophilic polyethersulfone membranes (Supor^®^, 0.45 µm, 25 mm diameter, Pall Corporation, Port Washington, NY, USA) served as the synthetic diffusion barrier. Before the experiments, the membranes were equilibrated in the receptor medium for 30 min.

The receptor compartment was filled with physiological saline solution containing 60% (*v*/*v*) ethanol, previously heated and degassed to ensure sink condition throughout the experiment. Approximately 0.300 g of each gelled formulation was uniformly applied to the membrane surface in the donor compartment, avoiding air entrapment. During the experiments, the diffusion cells were maintained at 32 ± 2 °C and the receptor medium was continuously stirred at 600 rpm. Release studies were conducted over 8 h. Aliquots (0.5 mL) were automatically withdrawn from the receptor compartment after 0.5, 1, 2, 3, 4, 5, 6, 7 and 8 h and immediately replaced with an equal volume of fresh receptor medium to maintain a constant volume and sink conditions. CEO content was quantified by UV–Vis spectrophotometry (T70+, PG Instruments Ltd., Leicestershire, UK) at 292 nm using an external calibration curve prepared in the receptor medium (0.316–6.324 µg mL^−1^, R^2^ = 0.9996). The analytical wavelength was selected based on the characteristic absorption of cinnamaldehyde, the principal constituent of the CEO used in this study. Each formulation was tested in five independent replicates.

*In vitro skin permeation studies*

*Preparation of skin membranes*

Excised porcine ear skin was selected as the biological membrane because of its well-established structural and permeability similarity to human skin. Skin samples were obtained from the ears of healthy 4-month-old pigs (both sexes) collected from a local slaughterhouse immediately after slaughter. The ears were rinsed with tap water, and the external surface was carefully shaved before skin excision. Split-thickness skin (approximately 500 µm) was prepared using an electric dermatome (Acculan^®^ 3 Ti Electrodermatome, Aesculap, B. Braun, Hazelwood, MO, USA). Freshly excised skin was used immediately or stored at −18 °C for up to 2 months. Before use, frozen samples were thawed at room temperature, visually inspected for structural integrity, and only undamaged specimens were selected for permeation experiments. The thickness of each membrane was measured using a digital micrometer before cutting into squares of approximately 2 cm^2^.

*Skin permeation experiments*

In vitro skin permeation studies were conducted using the same Franz diffusion cell setup as in the release experiments, with physiological saline containing 30% (*v*/*v*) ethanol as the receptor medium. Samples were collected at predetermined intervals up to 24 h and replaced with an equal volume of fresh medium after 1, 2, 3, 4, 5, 6, 8, 10, 12, 14, 16, 18, 20, 22 and 24 h and immediately replaced with an equal volume of fresh medium. CEO concentrations in the receptor fluid were determined by UV-Vis spectrophotometry at 292 nm using an external calibration curve constructed in receptor medium (0.372–7.44 µg mL^−1^; R^2^ = 0.9998). Each permeation experiment was performed using five independent replicates for each gelled CEO-loaded microemulsion formulation.

*Data analysis of in vitro release and skin permeation studies*

The cumulative amount of CEO released or permeated per unit membrane area (μg cm^−2^) was plotted as a function of time. From the linear portion of the permeation profiles, the steady-state flux (J_ss_, μg cm^−2^ h^−1^), permeability coefficient (K_P_, cm h^−1^) and release rate (k_H_, μg cm^−2^ h^−1/2^) were determined using the conventional diffusion equations.

To elucidate the release mechanism of CEO from the gelled microemulsions, the experimental release profiles were fitted to the zero-order (Equation (4)), first-order (Equation (5)), Higuchi (Equation (6)) and Korsmeyer–Peppas (Equation (7)) kinetic models:

(4) M_t_ = M_0_ + K_0_t

(5) logC = logC_0_ − K_1_t/2.303

(6) M = K_H_t^1/2^

(7) M_t_/M_∞_ = K_P_t^n^

In the above equations, M_t_ is the cumulative amount of CEO released at time t, M_∞_ is the total amount released at infinite time, K_0_, K_1_, K_H_ and K_P_ are the release rate constants corresponding to the respective kinetic models, t is the time, and *n* is the release exponent describing the predominant release mechanism.

Model fitting was performed by linear regression analysis, and the goodness of fit was assessed using the coefficient of determination (R^2^). The kinetic model providing the highest R^2^ value together with physically meaningful fitting parameters was considered the most appropriate for describing the release behavior of the investigated formulations.

#### 2.2.11. Statistical Analysis

Experimental data are presented as mean ± standard deviation (SD). Statistical comparisons among the three CEO-loaded formulations were performed using one-way analysis of variance (ANOVA), followed, when a significant overall effect was detected, by Tukey’s honestly significant difference (HSD) post hoc test for multiple pairwise comparisons. For datasets comprising both blank and corresponding CEO-loaded gelled microemulsions, two-way ANOVA was used to evaluate the effects of the decyl glucoside-to-propylene glycol (DG:PG) ratio, CEO incorporation, and their interaction. Where appropriate, Tukey’s HSD post hoc test was subsequently used for multiple pairwise comparisons. Statistical significance was set at *p* < 0.05. The receptor-medium solubility screening was evaluated descriptively against the predefined three-fold and ten-fold sink-condition criteria and was therefore not subjected to inferential statistical comparison. Linear and nonlinear regression analyses were performed for calibration curves, rheological modelling, and release and permeation kinetic modelling. Statistical analyses were performed using Microsoft Excel (Microsoft Office 2016, Microsoft Corporation, Redmond, WA, USA). Linear and nonlinear regression analyses were performed using the software packages specified for the respective experimental techniques, and, where appropriate, Microsoft Excel.

For the statistical evaluation of short-term stability, data obtained immediately after preparation (Day 0) and after 15, 30, and 45 days of storage were included in the analysis. Since the same samples were monitored longitudinally throughout the storage period, time-dependent changes in quantitative parameters were evaluated separately for each formulation using one-way repeated-measures ANOVA, with storage time as the within-sample factor. Where a significant overall effect of storage time was identified, paired *t*-tests comparing each storage time point with baseline were performed, with Holm adjustment for multiple comparisons. Statistical significance was set at *p* < 0.05.

## 3. Results

### 3.1. Selection of the Microemulsion Composition

#### 3.1.1. Selection of the Oil Phase

A binary oil phase consisting of CEO and IPM was selected for microemulsion formulation. The mass ratio of CEO and IPM in the binary mixture was 2:1. The final formulation contained 3% (*w*/*w*) CEO.

#### 3.1.2. Selection of the S_mix_ Composition

Decyl glucoside was selected as the surfactant for the development of CEO-loaded cutaneous microemulsions. Propylene glycol (PG) and diethylene glycol monoethyl ether (DEGEE) were comparatively evaluated as cosurfactants because of their different physicochemical properties, which may influence microemulsion formation and composition. Accordingly, pseudo-ternary phase diagrams were constructed using both surfactant/cosurfactant mixtures to determine the most suitable S_mix_ composition for further formulation development.

#### 3.1.3. Pseudo-Ternary Phase Diagrams: Effect of Cosurfactant and S_mix_ Ratio on Microemulsion Formation

**Figure 1:**
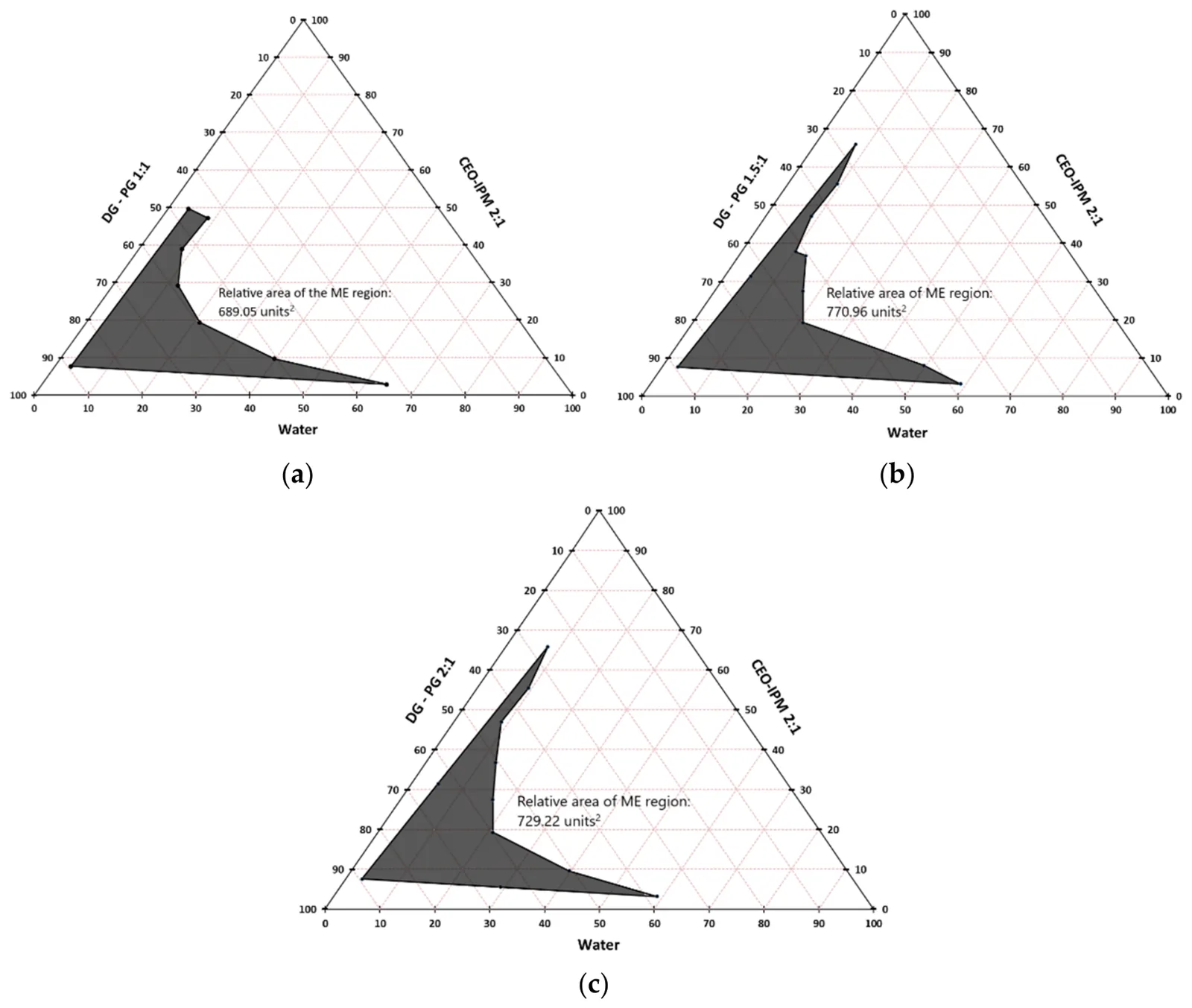
Pseudo-ternary phase diagrams of the CEO/IPM (2:1, *w*/*w*)-DG/PG-water systems constructed at different decyl glucoside (DG) to propylene glycol (PG) mass ratios: (**a**) 1:1, (**b**) 1.5:1, and (**c**) 2:1.

**Figure 2:**
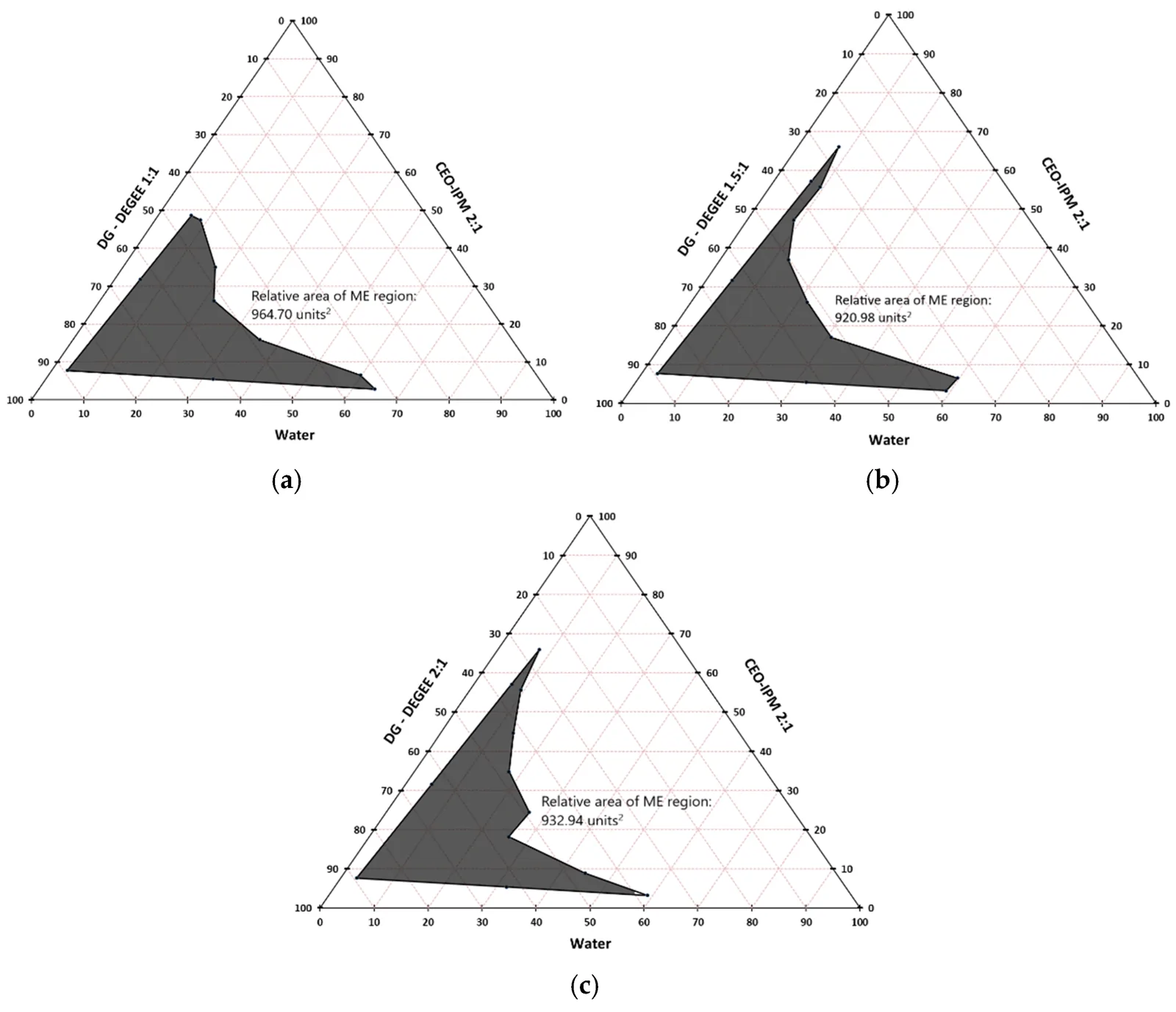
Pseudo-ternary phase diagrams of the CEO/IPM (2:1, *w*/*w*)-DG/DEGEE-water systems constructed at different decyl glucoside (DG) to diethylene glycol monoethyl ether (DEGEE) mass ratios: (**a**) 1:1, (**b**) 1.5:1, and (**c**) 2:1.

Pseudo-ternary phase diagrams were constructed to evaluate the effects of cosurfactant type and surfactant-to-cosurfactant mass ratio on the formation of microemulsions containing CEO/IPM oil phase (2:1, *w*/*w*), decyl glucoside (DG), and water. Propylene glycol (PG) and diethylene glycol monoethyl eter (DEGEE) were comparatively investigated as cosurfactants at DG:cosurfactant ratios of 1:1, 1.5:1, and 2:1. The dark-shaded areas in Figure 1 and Figure 2 represent the transparent, isotropic microemulsion domains identified by aqueous titration.

The phase behavior was strongly dependent on the nature of the cosurfactant. At each investigated S_mix_ ratio, DEGEE generated a broader microemulsion region than PG. At DG:cosurfactant ratios of 1:1, 1.5:1, and 2:1, the microemulsion areas increased from 689.05, 770.96, and 729.22 units^2^ for the PG-based systems to 964.70, 920.98, and 932.94 units^2^ for the corresponding DEGEE-based systems, respectively. These differences represent approximate increases of 40.0%, 19.5%, and 27.9%, indicating that DEGEE provided a wider compositional window for microemulsion formation throughout the investigated range of S_mix_ ratios.

The influence of the DG:cosurfactant ratio differed between the two series. In the PG-containing systems, increasing the DG:PG ratio from 1:1 to 1.5:1 enlarged the microemulsion domain from 689.05 to 770.96 units^2^, whereas a further increase to 2:1 reduced the area to 729.22 units^2^. Thus, the largest PG-based microemulsion region was obtained at a DG:PG ratio of 1.5:1.

#### 3.1.4. Selection of the Final Formulations

Propylene glycol was selected as the cosurfactant for subsequent formulation studies. The concentration of CEO in the final microemulsion formulations selected from the pseudoternary phase diagrams constructed for the CEO/IPM-DG/PG-water systems was fixed at 3% (*w*/*w*). Since the CEO:IPM ratio was maintained at 2:1 (*w*/*w*), the selected CEO concentration resulted in a total oil phase content of 4.5% (*w*/*w*). Within each pseudo-ternary phase diagram, formulations containing 45% (*w*/*w*) S_mix_ were selected.

### 3.2. Physicochemical Characterization of O/W Cinnamon Bark Essential Oil-Loaded Microemulsions

**Figure 3:**
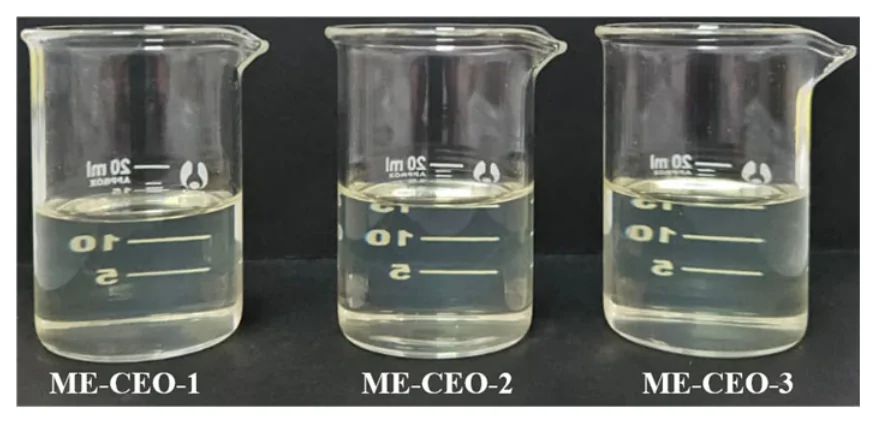
Macroscopic appearance of the selected O/W CEO-loaded microemulsions immediately after preparation.

The macroscopic appearance of the three selected hydrophilic CEO-loaded microemulsions is presented in Figure 3.

All formulations appeared as clear, transparent, and visually homogeneous liquids, with no evidence of phase separation, turbidity, sedimentation, or creaming immediately after preparation. The systems exhibited a pale-yellow color, attributable to the natural color of CEO, and retained their characteristic aromatic odor.

All formulations exhibited low viscosity and readily flowed upon tilting the container, confirming their liquid character.

No visual differences were observed among the three formulations despite the different surfactant-to-cosurfactant ratios.

The characteristic odor of CEO was preserved in all formulations, with no detectable off-odors.

The selected O/W CEO-loaded microemulsions were further characterized in terms of droplet size and size distribution, zeta potential, dynamic viscosity, refractive index, and apparent pH (Table 2).

All three formulations displayed nanometric mean droplet diameters, ranging from 24.95 ± 0.35 to 34.62 ± 0.18 nm (Table 2). A progressive decrease in mean droplet diameter was observed from ME-CEO-1 to ME-CEO-3, concomitant with the increase in decyl glucoside concentration from 30% (*w*/*w*) to 36.0% (*w*/*w*) and the corresponding decrease in propylene glycol content. ME-CEO-3 exhibited the smallest droplets (24.95 ± 0.35 nm), whereas ME-CEO-1 showed the largest mean diameter (34.62 ± 0.18 nm).

The PDI values ranged from 0.136 ± 0.023 to 0.312 ± 0.016 (Table 2). ME-CEO-1 and ME-CEO-3 exhibited narrow droplet size distributions, with PDI values of 0.144 ± 0.024 and 0.136 ± 0.023, respectively. ME-CEO-2 presented a broader, although still acceptable, distribution (PDI = 0.312 ± 0.016).

The mean droplet diameter differed significantly among the three formulations (*p* < 0.001), with significant pairwise differences between all formulations (Tukey’s test, *p* < 0.05). PDI also differed significantly among formulations (*p* < 0.001), with ME-CEO-2 exhibiting a significantly higher value than ME-CEO-1 and ME-CEO-3, whereas no significant difference was observed between ME-CEO-1 and ME-CEO-3.

**Figure 4:**
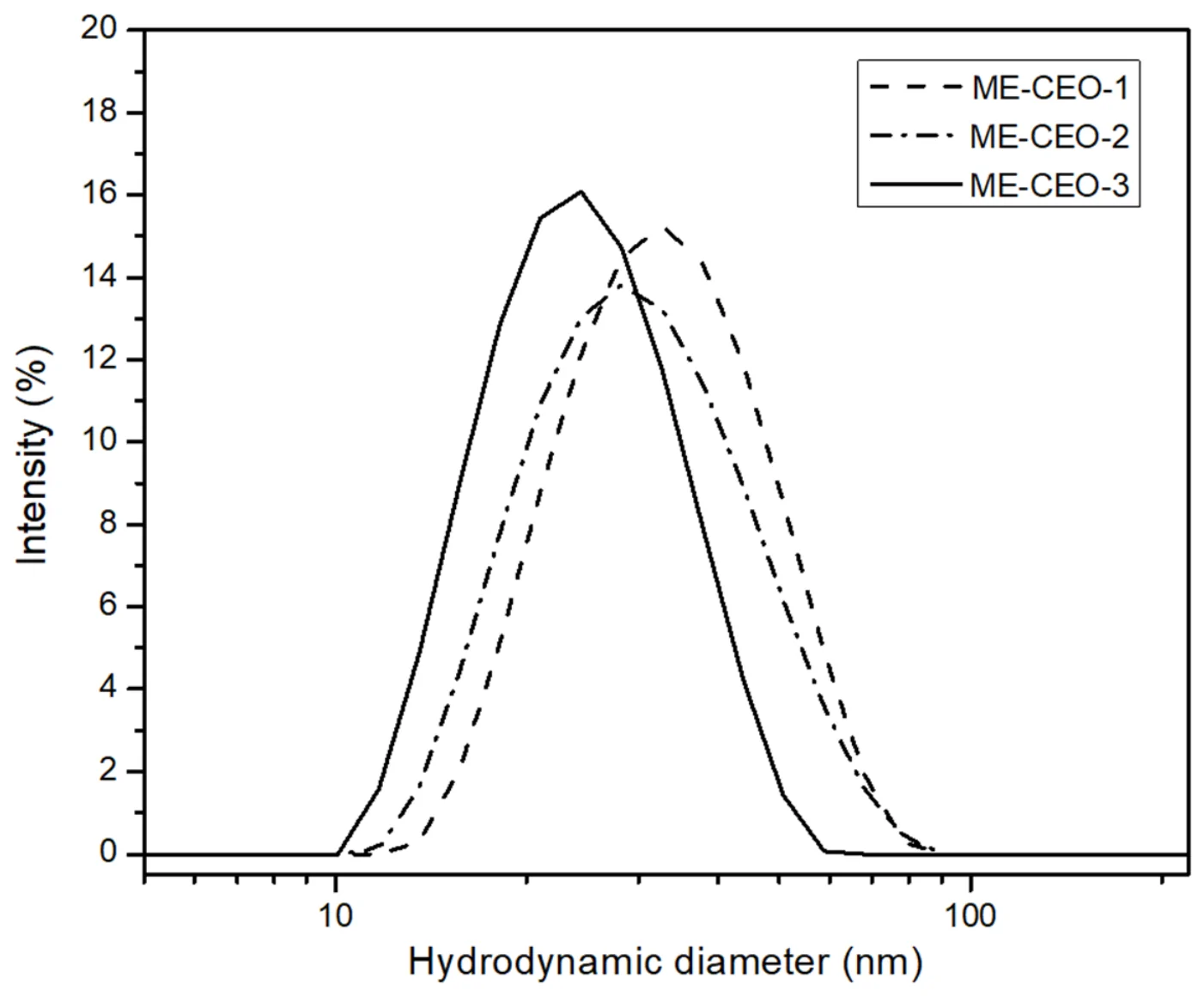
Intensity-based droplet size distribution profiles of the studied CEO-loaded microemulsions determined by dynamic light scattering. Data shown as the mean of three independent measurements.

The intensity-based droplet size distributions are presented in Figure 4.

All formulations exhibited single, narrow, unimodal distribution profiles without evidence of secondary particle populations or aggregation. A progressive shift in the distribution towards smaller diameters was observed as the DG:PG ratio increased, consistent with the reduction in mean droplet size determined by DLS. ME-CEO-3 exhibited the leftmost distribution profile, whereas ME-CEO-1 showed the largest droplet population. Despite these differences, the substantial overlap among the three curves indicates that all formulations possessed comparable nanoscale organization.

All formulations presented slightly negative, near-neutral zeta potential values, ranging from − 0.927 ± 0.24 to −0.726 ± 0.28 mV (Table 2), with no significant differences among the three formulations (*p* > 0.05).

The refractive indices were similar, ranging from 1.381 ± 0.002 to 1.383 ± 0.003 (Table 2), and followed a slight decreasing trend from ME-CEO-1 to ME-CEO-3.

The studied microemulsion systems were low-viscosity liquids, with dynamic viscosity values between 26 ± 1.82 and 28 ± 3.27 mPa·s (Table 2). Modification of the DG:PG ratio within the investigated interval had no pronounced effect on the bulk viscosity of the systems. In contrast to mean droplet diameter and PDI, dynamic viscosity, refractive index, and zeta potential did not differ significantly among the three formulations (*p* > 0.05).

Following adjustment with citric acid, all formulations exhibited mildly acidic apparent pH values within a narrow interval of 5.59 ± 0.001 to 5.65 ± 0.009 (Table 2). Although the apparent pH values were confined to a narrow mildly acidic range, statistically significant differences were detected among the three formulations (*p* < 0.001). No systematic dependence of final apparent pH on the DG:PG ratio was evident.

### 3.3. Characterization of Gelled Cinnamon Bark Essential Oil-Loaded Microemulsions

**Figure 5:**
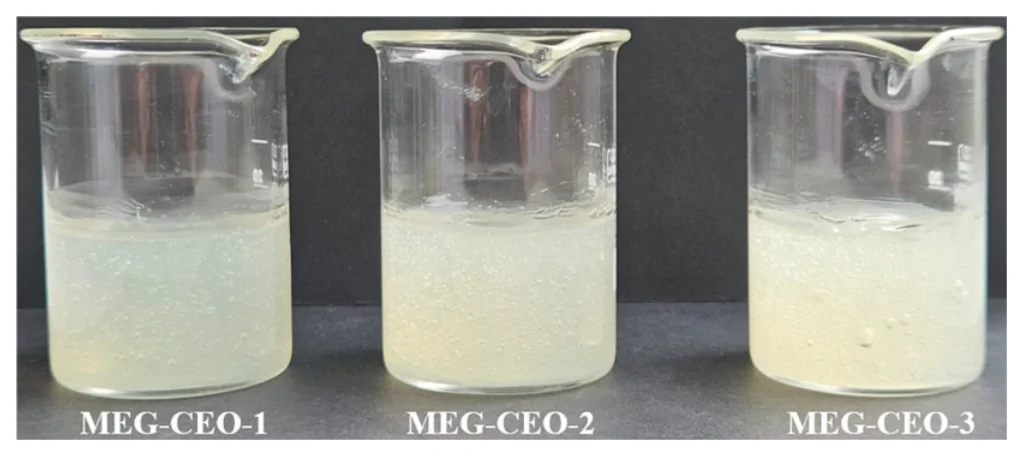
Macroscopic appearance of the gelled CEO-loaded microemulsions immediately after preparation.

The macroscopic appearance of the three Aerosil^®^ 200 gelled CEO-loaded microemulsions is presented in Figure 5.

Following gelation with 4% (*w*/*w*) Aerosil^®^ 200, all CEO-loaded formulations remained visually homogeneous, with no evidence of phase separation, oil leakage, sedimentation, or syneresis immediately after preparation (Figure 5). Compared with the corresponding liquid microemulsions (Figure 3), which were clear and transparent, the gelled systems became translucent to slightly opaque, while retaining the pale-yellow hue imparted by the CEO. This visual change may be related to the formation of the silica network and increased light scattering within the system.

All gelled formulations showed a semisolid consistency and no longer flowed freely upon tilting the container, confirming the successful development of a continuous gel network. The incorporation of Aerosil^®^ 200 transformed the low-viscosity microemulsions into semisolid formulations, while maintaining the physicochemical characteristics of the original microemulsion systems and primarily altering their rheological properties. No appreciable macroscopic differences were observed among MEG-CEO-1, MEG-CEO-2 and MEG-CEO-3, despite the different surfactant-to-cosurfactant ratios used in the parent microemulsions. The similar appearance of the gelled formulations suggests that variations in the initial microemulsion composition did not noticeably influence the gelation process or the overall macroscopic characteristics after incorporation of Aerosil^®^ 200.

The characteristic odor of CEO was preserved in all formulations, with no detectable off-odors, indicating that the gelation process did not induce perceptible organoleptic changes or signs of essential oil degradation.

#### Determination of Cinnamon Bark Essential Oil Content and Apparent pH

The CEO content and apparent pH values of the experimental gelled microemulsions are presented in Table 3.

The spectrophotometric assay demonstrated excellent retention of the essential oil during the gelation process. The measured CEO contents ranged from 99.41 ± 0.81% to 100.78 ± 0.43% of the theoretical value, corresponding to deviations below 1% from the nominal content. CEO content showed no statistically significant differences among the three gelled formulations (one-way ANOVA, *p* = 0.210), confirming comparable CEO loading across the investigated systems. Following gelation and adjustment with 20% (*w*/*w*) aqueous citric acid solution, all formulations exhibited mildly acidic apparent pH values ranging from 6.14 ± 0.006 to 6.35 ± 0.006. The apparent pH differed significantly among all three gelled formulations (*p* < 0.001), decreasing from MEG-CEO-1 to MEG-CEO-3.

### 3.4. Rheological Analysis

#### 3.4.1. Steady-Shear State Measurements

**Figure 6:**
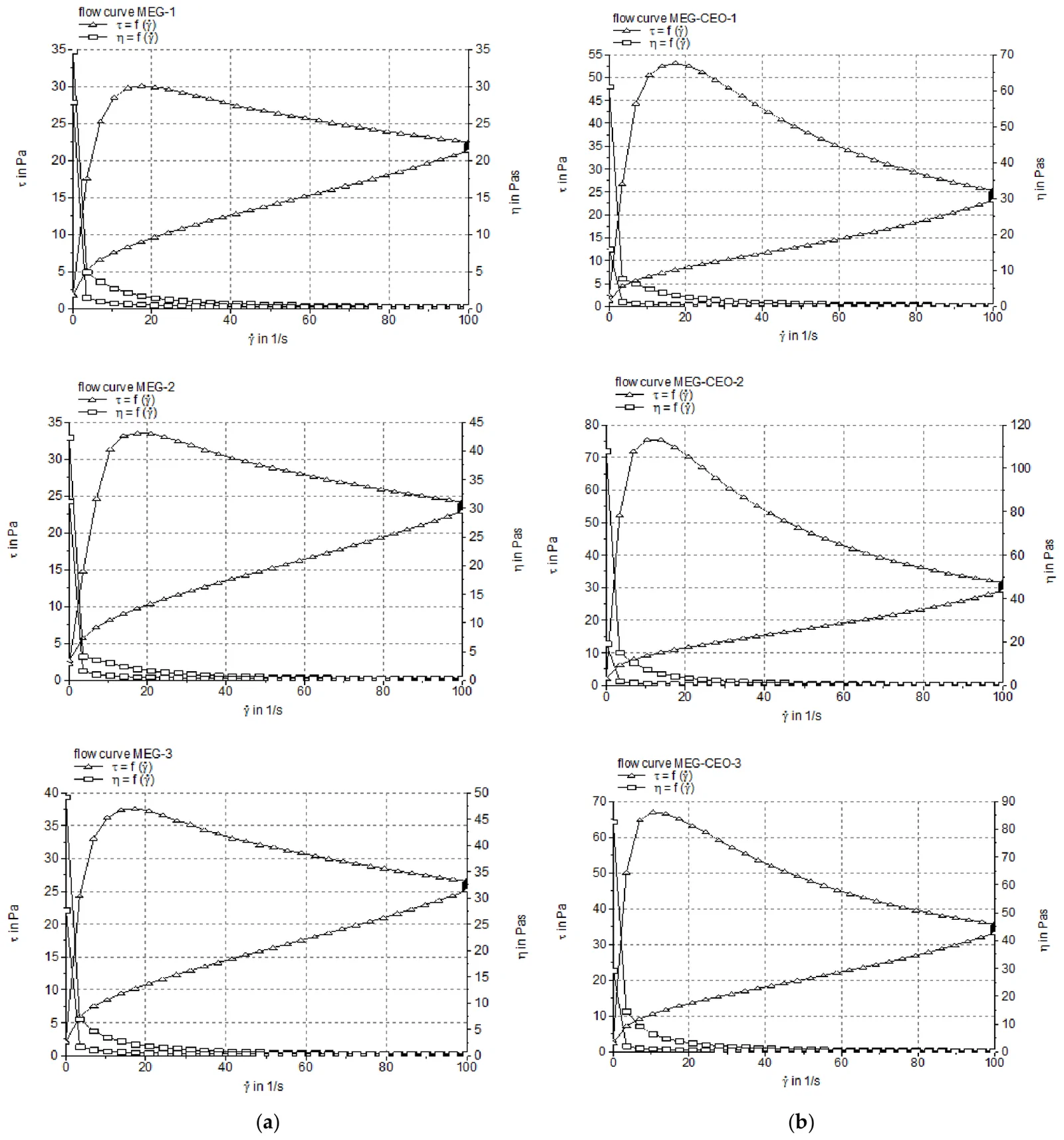
Flow and viscosity profiles of the experimental Aerosil^®^200-gelled blank microemulsions (MEG-1 to MEG-3; column (**a**) and the corresponding CEO-loaded formulations (MEG-CEO-1 to MEG-CEO-3; column (**b**).

The steady-state flow curves and apparent viscosity profiles of the gelled CEO-loaded microemulsions and the corresponding blank formulations are presented in Figure 6, whereas the apparent viscosity and thixotropy index values are summarized in Table 4.

Apparent viscosity was significantly influenced by both the DG:PG ratio and CEO incorporation (two-way ANOVA, *p* < 0.001 for both factors), with a significant interaction between the two factors (*p* < 0.001). Within both the blank and CEO-loaded series, apparent viscosity increased progressively from formulation 1 to formulation 3. CEO incorporation significantly increased apparent viscosity relative to the corresponding blank formulation at each DG:PG ratio (*p* < 0.05), with increases of approximately 1.11-, 1.29-, and 1.33-fold for MEG-CEO-1, MEG-CEO-2, and MEG-CEO-3, respectively.

#### 3.4.2. Small-Amplitude Oscillatory Measurements

**Figure 7:**
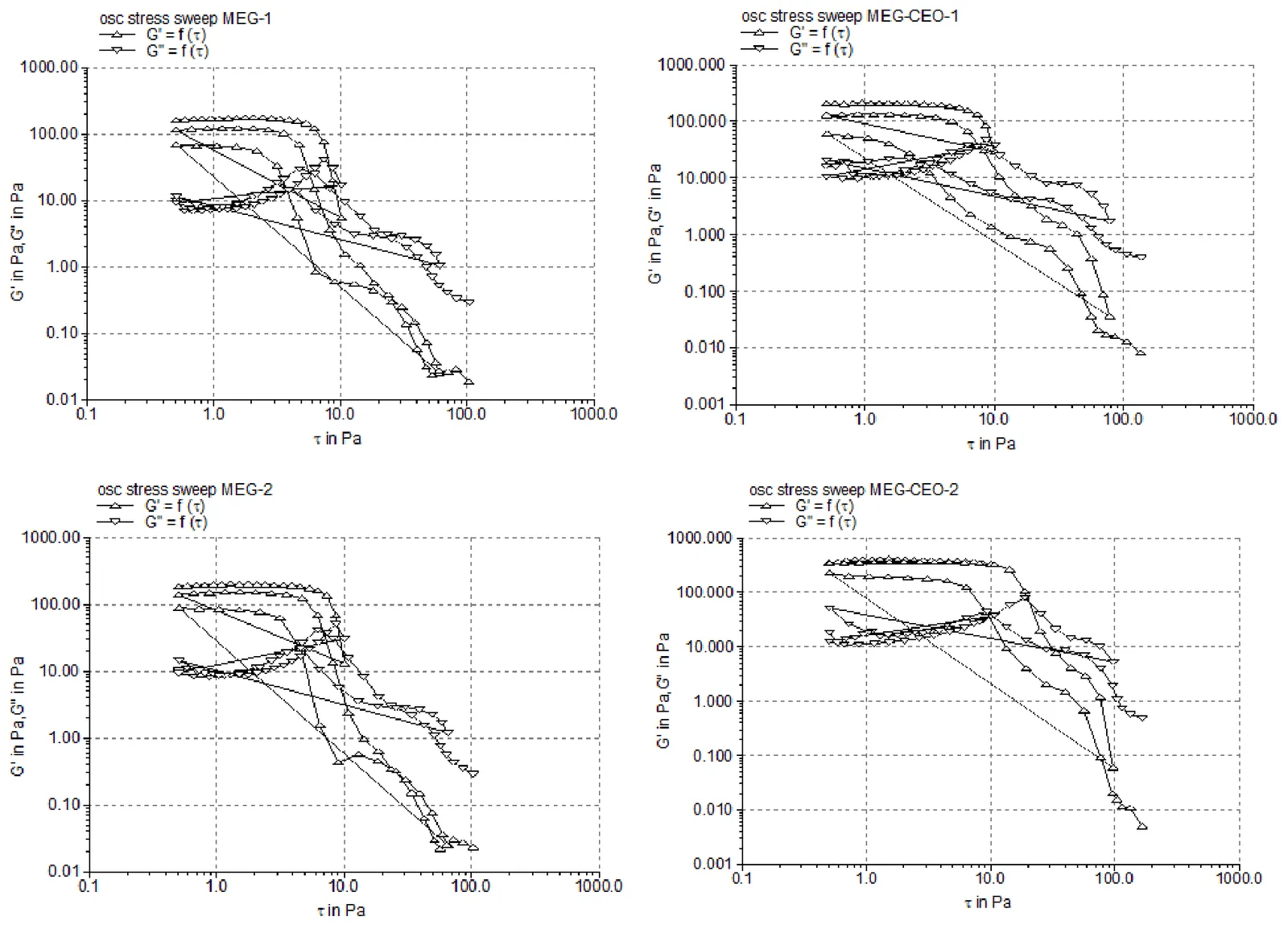

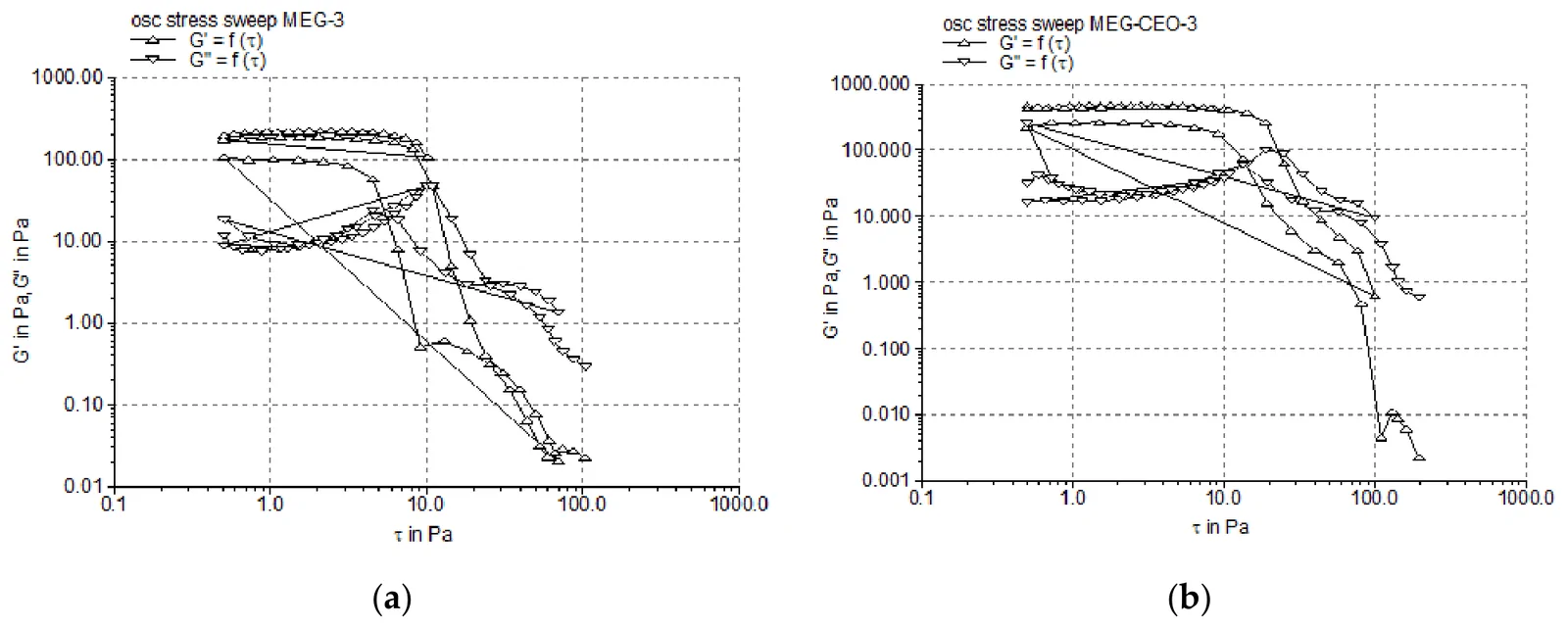
Oscillatory stress sweep profiles of the gelled blank microemulsions (MEG-1 to MEG-3; column (**a**) and the corresponding CEO-loaded formulations (MEG-CEO-1 to MEG-CEO-3; column (**b**). Storage modulus (G’) and loss modulus (G”) were recorded as a function of the applied oscillatory shear stress at a constant frequency of 1.00 Hz to determine the linear viscoelastic region (LVR).

The viscoelastic properties of the gelled microemulsions were investigated by oscillatory stress sweep measurements performed at a constant frequency of 1.00 Hz. Representative stress sweep profiles are presented in Figure 7, while the corresponding values of the storage modulus (G’), loss modulus (G”), and loss factor (tan δ) measured within the linear viscoelastic region (LVR) are summarized in Table 5.

Two-way ANOVA demonstrated that G’ was significantly affected by both DG:PG ratio and CEO incorporation, with a significant interaction between these factors (*p* < 0.001 in all cases). CEO incorporation significantly increased G’ relative to the corresponding blank formulation at each DG:PG ratio, with increases of approximately 1.22-, 1.99-, and 2.17-fold for MEG-CEO-1, MEG-CEO-2, and MEG-CEO-3, respectively. G” was also significantly affected by DG:PG ratio (*p* = 0.008), CEO incorporation (*p* < 0.001), and their interaction (*p* = 0.013), although pairwise differences between CEO-loaded and corresponding blank formulations were significant only for the third formulation pair. Yield stress showed a significant overall effect of CEO incorporation (*p* = 0.020), whereas the effect of DG:PG ratio and the interaction between the two factors were not significant (*p* > 0.05).

#### 3.4.3. Frequency Sweep Measurements

**Figure 8:**
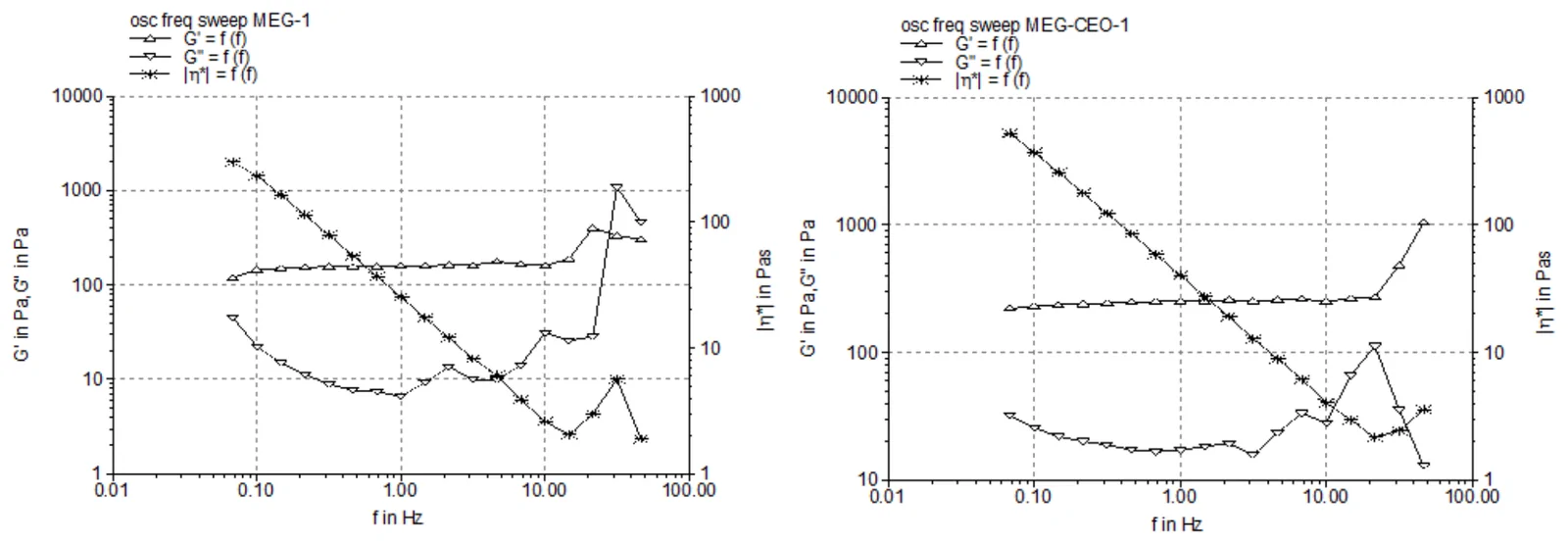

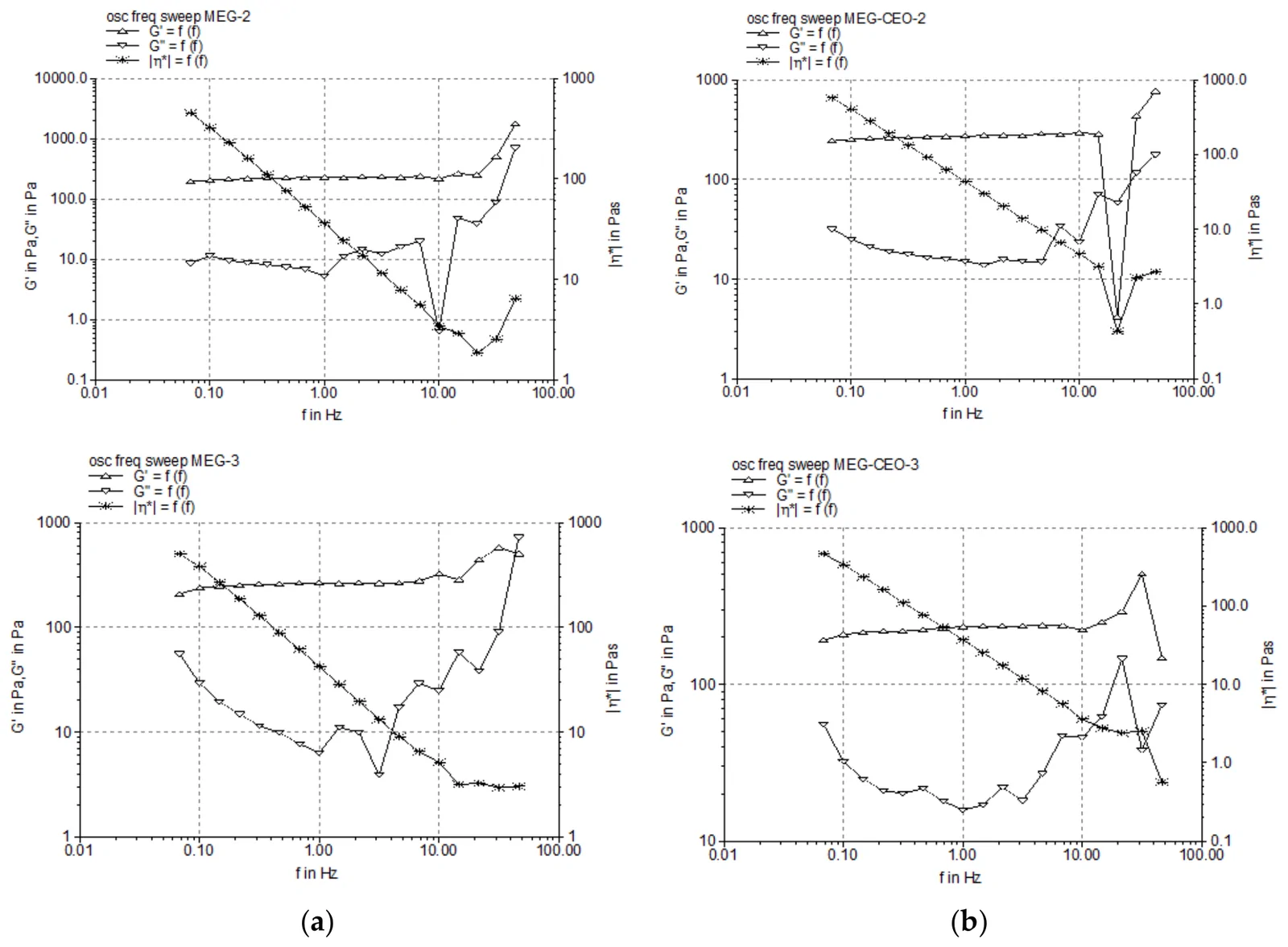
Oscillatory frequency sweep profiles of the gelled blank microemulsions (MEG-1 to MEG-3, column (**a**) and the corresponding CEO-loaded formulations (MEG-CEO-1 to MEG-CEO-3, column (**b**). Storage modulus (G’), loss modulus (G”) and complex viscosity (η*) were recorded over a frequency range of 0.05–50 Hz at a constant oscillatory shear stress of 1 Pa selected within the linear viscoelastic region.

The frequency dependence of the viscoelastic behavior of the gelled microemulsions was further evaluated by oscillatory frequency sweep measurements. Based on the preceding stress sweep measurements, a constant oscillatory stress of 1 Pa, located within the LVR of all investigated formulations, was selected for the frequency sweep measurements to ensure non-destructive testing conditions. The resulting profiles of the storage modulus (G’), loss modulus (G”) and complex viscosity (η*) are presented in Figure 8. The deviations observed at the highest oscillation frequencies (20–50 Hz) are most likely attributable to instrumental inertia and therefore were not considered for structural interpretation.

### 3.5. Consistency Measurements

**Figure 9:**
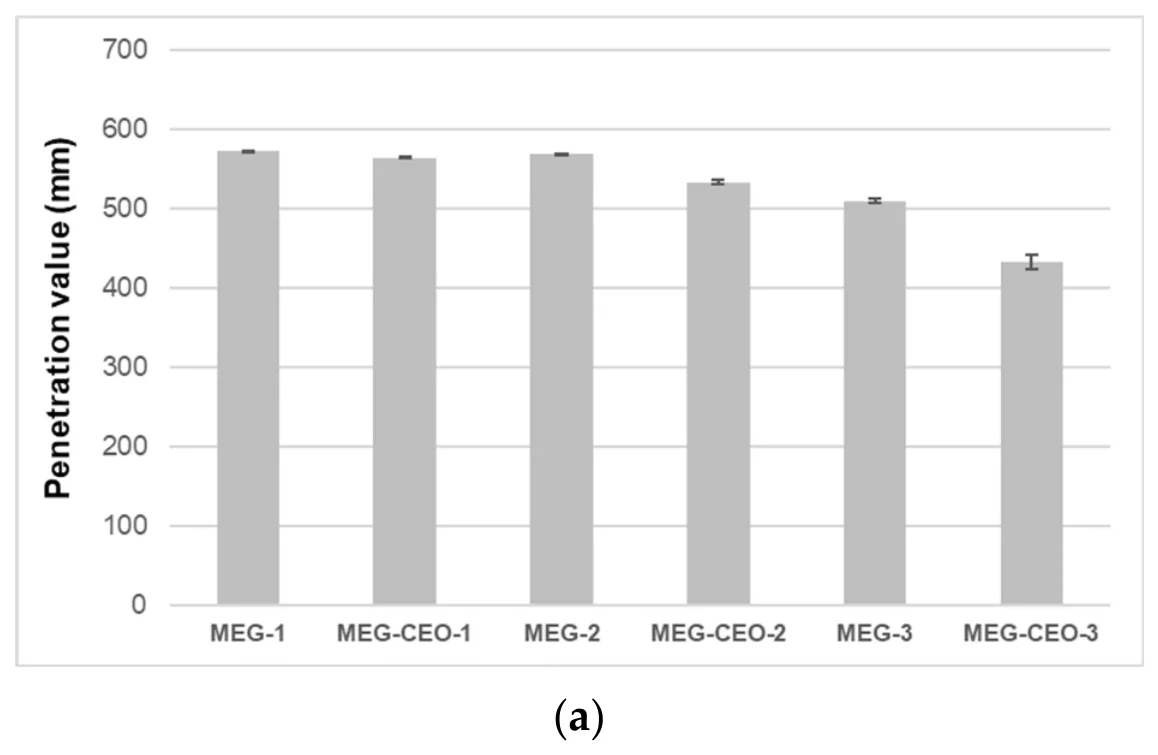

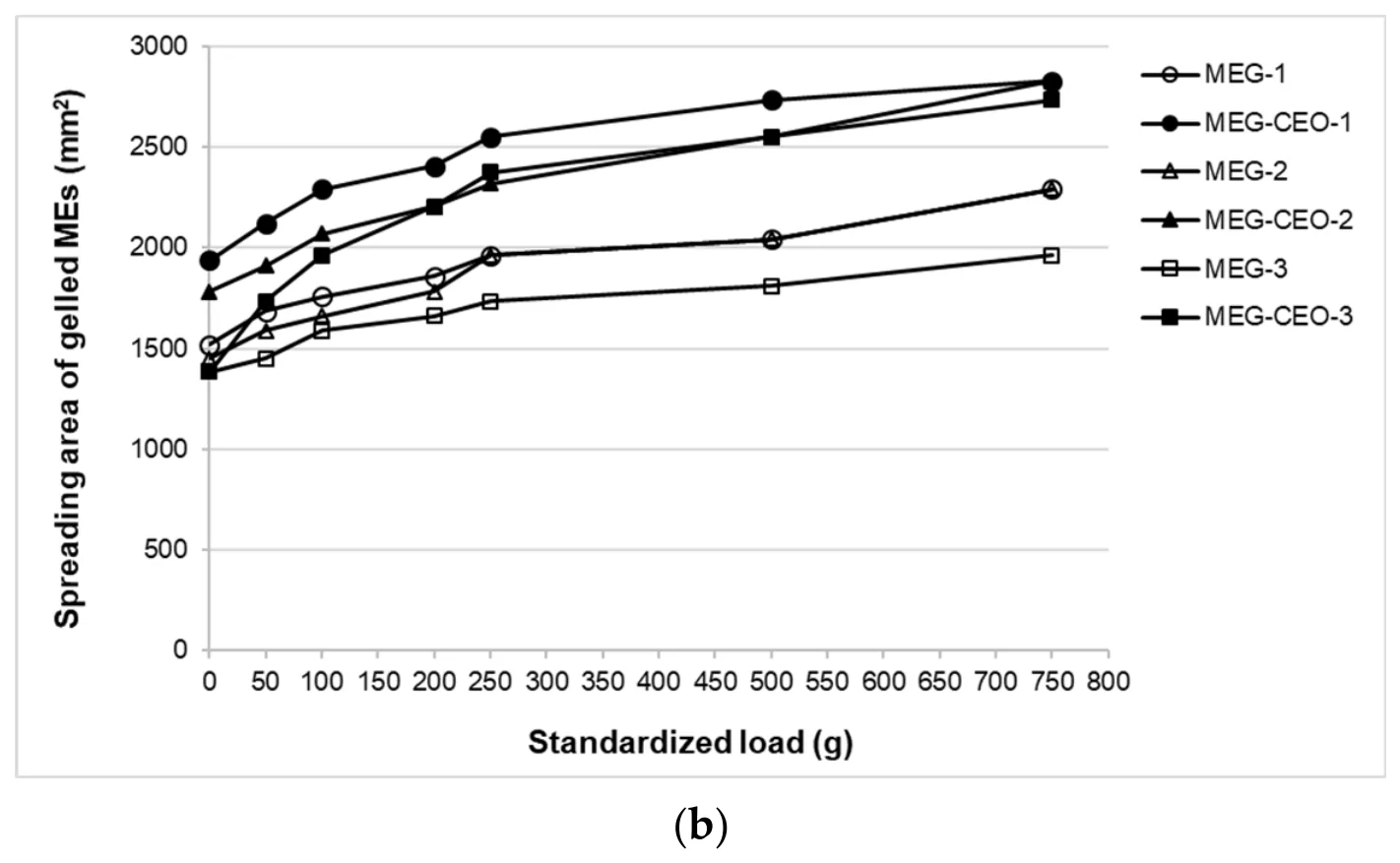
Mechanical characterization of the gelled microemulsions. (**a**) Penetration values determined by the pharmacopeial penetrometric method. (**b**) Spreadability profiles obtained by the parallel-plate method (mean ± SD, *n* = 3).

The consistency of the gelled formulations was evaluated using two complementary methods, namely penetrometry and parallel-plate spreadability test (Figure 9).

In comparison with corresponding blank formulations, penetration values decreased by approximately 1%, 6% and 15% for MEG-CEO-1, MEG-CEO-2 and MEG-CEO-3, indicating progressively higher formulation hardness (Figure 9). The effect became more pronounced with increasing surfactant concentration, with the lowest penetration value observed for MEG-CEO-3 (432 ± 5.83 mm). The highest penetration values were recorded for the blank formulations MEG-2 (568 ± 3.41) and MEG-1 (572 ± 0.82). Since lower penetration values reflect greater resistance to probe penetration, these results demonstrate that CEO incorporation increased the mechanical strength of the gel matrix.

### 3.6. Texture Analysis

**Figure 10:**
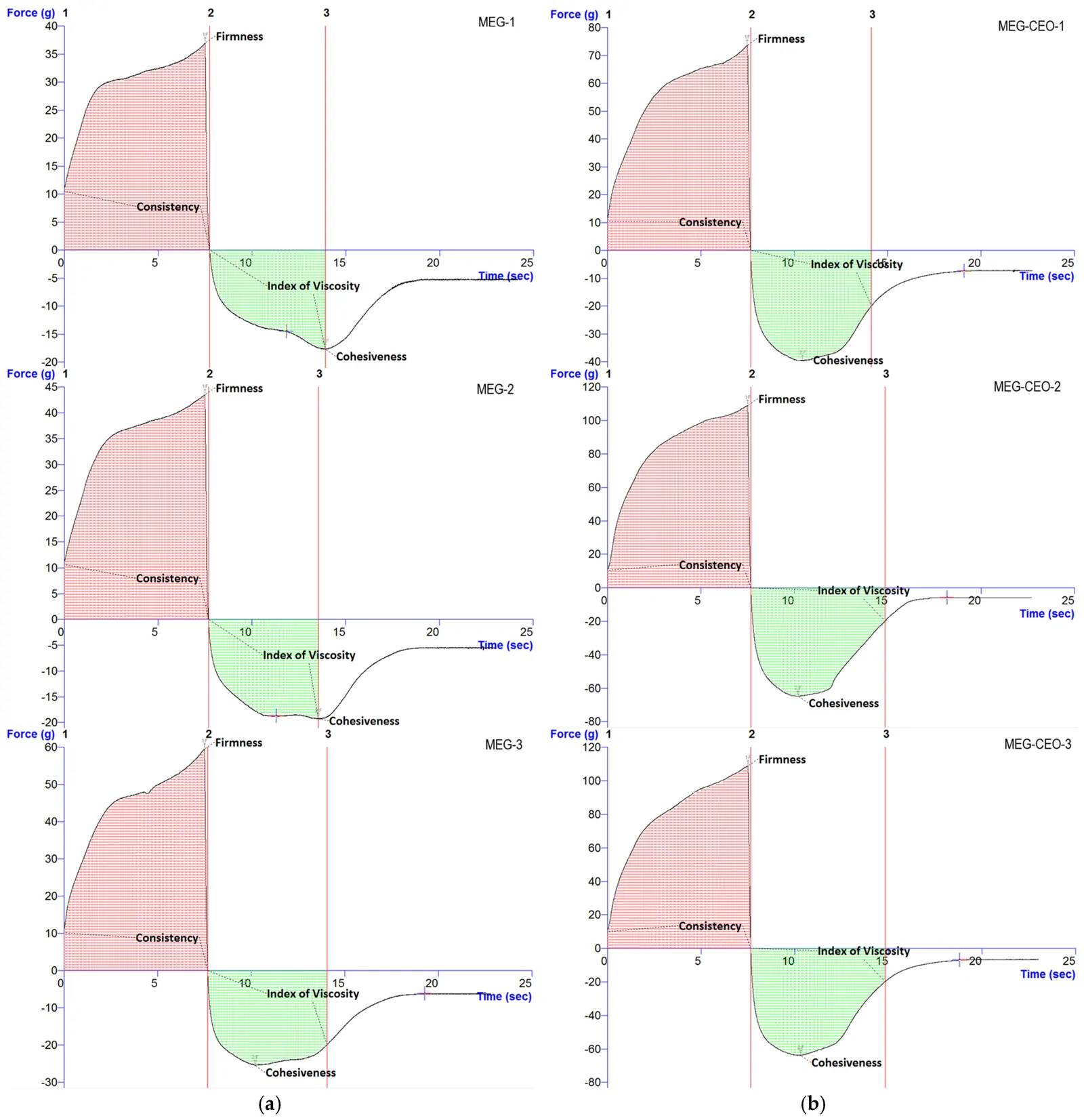
Representative force–time curves obtained during the back extrusion test for blank gelled microemulsions (MEG-1 to MEG-3, column (**a**) and the corresponding CEO-loaded formulations (MEG-CEO-1 to MEG-CEO-3, column (**b**). The positive (red) and negative (green) areas of the force-time curves were used to calculate firmness and consistency, respectively, and the cohesiveness and viscosity index. The red vertical lines indicate the integration limits used for calculating the viscosity index.

**Figure 11:**
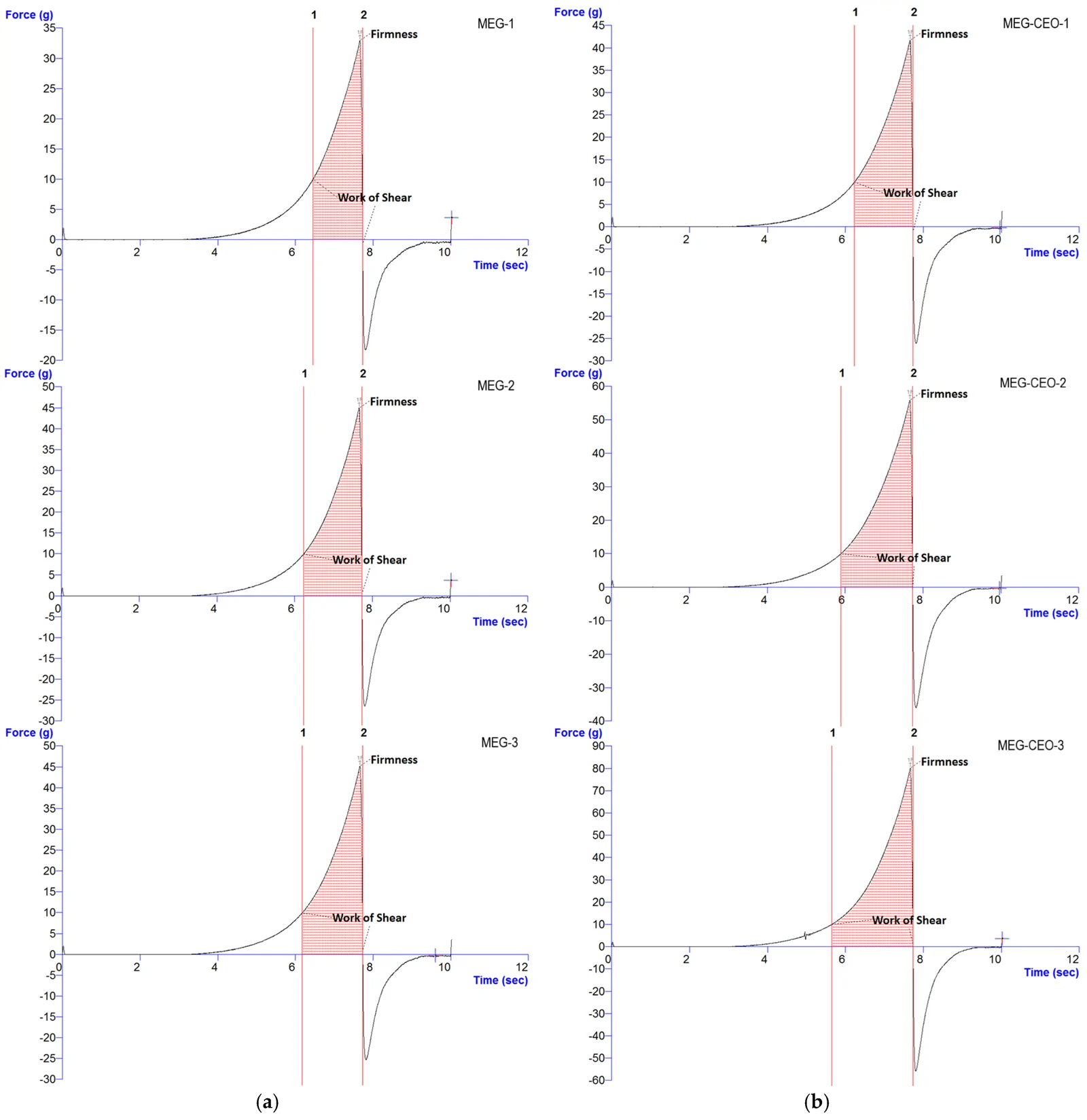
Representative force–time curves obtained during the spreadability test for blank gelled microemulsions (MEG-1 to MEG-3, column (**a**) and the corresponding CEO-loaded formulations (MEG-CEO-1 to MEG-CEO-3, column (**b**). The positive (red) area of the force-time curves was used to calculate firmness and work of shear.

Representative force-time curves obtained during the back extrusion and spreadability tests are presented in Figure 10 and Figure 11, whereas the calculated textural parameters are summarized in Table 6 and Table 7. The texture profiles revealed clear differences in the mechanical behavior of the blank and CEO-loaded gelled microemulsions, confirming the influence of CEO on the structural properties of the formulations.

Two-way ANOVA demonstrated significant effects of both DG:PG ratio and CEO incorporation on all back-extrusion parameters, with significant interactions between the two factors (*p* < 0.05). CEO incorporation significantly increased firmness and consistency and increased the absolute magnitude of cohesiveness and viscosity index relative to the corresponding blank formulation at all three DG:PG ratios (Tukey’s test, *p* < 0.05). Among the CEO-loaded systems, MEG-CEO-2 and MEG-CEO-3 exhibited significantly greater firmness, cohesiveness magnitude, and viscosity-index magnitude than MEG-CEO-1, whereas no significant differences were detected between MEG-CEO-2 and MEG-CEO-3 for these parameters. Consistency differed significantly among all three CEO-loaded formulations, with the highest value recorded for MEG-CEO-2.

The spreadability test showed that CEO incorporation increased the firmness of all formulations. Compared with the corresponding blank gels, firmness increased by approximately 1.3-fold for MEG-CEO-1 and MEG-CEO-2 and by 1.8-fold for MEG-CEO-3, indicating greater resistance to the initiation of spreading. The work of shear presented the same tendency, increasing by approximately 1.4-fold for MEG-CEO-1 and MEG-CEO-2 and by 1.9-fold for MEG-CEO-3. Since work of shear reflects the total mechanical energy required to spread the formulation, these results demonstrate that CEO incorporation increased the resistance of the gel matrix throughout the spreading process. Furthermore, two-way ANOVA revealed significant effects of both DG:PG ratio and CEO incorporation on firmness and work of shear, together with significant interactions between the two factors (*p* < 0.001). CEO incorporation significantly increased both parameters relative to the corresponding blank formulations at all three DG:PG ratios (Tukey’s test, *p* < 0.05). Among the CEO-loaded formulations, firmness and work of shear increased significantly from MEG-CEO-1 to MEG-CEO-3, with significant differences between all three formulations. In contrast, among the blank systems, MEG-2 and MEG-3 did not differ significantly for either parameter. The particularly high values recorded for the MEG-CEO-3 system are consistent with its higher storage modulus, yield stress, penetration resistance and back-extrusion parameters, confirming the greater mechanical strength of this formulation.

### 3.7. Preliminary Short-Term Physicochemical Stability Assessment of Cinnamon Bark Essential Oil-Loaded Microemulsions and Corresponding Gelled Microemulsions

The macroscopic appearance of both CEO-loaded microemulsions and gelled microemulsions remained unchanged throughout the 45-day storage period. All liquid microemulsions maintained their initial transparent, homogeneous, pale-yellow appearance, without phase separation, sedimentation, or creaming. Similarly, the gelled formulations retained their homogeneous semisolid appearance, with no visible signs of physical instability. The physicochemical parameters determined at Days 15, 30, and 45 are summarized in Table 8 and Table 9 for the liquid and gelled microemulsions, respectively.

One-way repeated-measures ANOVA revealed no statistically significant time-dependent changes in droplet diameter, polydispersity index, dynamic viscosity, zeta potential, or apparent pH for any of the liquid microemulsions (*p* > 0.05). Similarly, no significant variations in CEO content, apparent viscosity, or penetration values were detected for any of the gelled formulations during storage (*p* > 0.05). Apparent pH also remained statistically unchanged for MEG-CEO-1 and MEG-CEO-3 (*p* > 0.05). For MEG-CEO-2, a significant overall effect of storage time on apparent pH was identified (*p* = 0.035). However, subsequent paired comparisons between Day 0 and each storage time point, with Holm adjustment for multiple comparisons, revealed no statistically significant differences (adjusted *p* > 0.05). The mean apparent pH values ranged from 6.236 ± 0.023 to 6.270 ± 0.005 throughout the observation period, with the value recorded at Day 45 (6.265 ± 0.013) remaining close to the baseline value (6.270 ± 0.005).

### 3.8. In Vitro Cinnamon Bark Essential Oil Release and Permeation Studies

#### 3.8.1. Selection of the Receptor Media for In Vitro Release and Permeation Studies

The apparent solubility of CEO increased markedly with increasing ethanol concentration irrespective of the aqueous phase employed (Table 10).

In both PBS- and saline-based media, raising the ethanol content from 30 to 70% (*v*/*v*) produced a substantial reduction in the volume of receptor medium required to dissolve 0.1 mL CEO (Table 10), indicating a pronounced improvement in solubilizing capacity. This behavior reflects the progressive reduction in the polarity of the receptor medium, which enhances the solubilization of the predominantly hydrophobic constituents of CEO. At identical ethanol concentrations, normal saline solution consistently exhibited higher apparent CEO solubility than PBS (pH 7.4). This behavior can be attributed to differences in the ionic composition of the aqueous phase. The phosphate salts present in the PBS can slightly reduce the solubilizing capacity of the hydroethanolic medium for the hydrophobic constituents of CEO through electrolyte-induced effects, whereas normal saline provides a simpler ionic environment with fewer interactions affecting the solvent properties. Nevertheless, ethanol concentration remained the dominant factor governing CEO solubilization.

Comparison of the experimental apparent solubility values with the calculated sink condition requirements showed that PBS containing 30% ethanol (*v*/*v*) did not provide sufficient CEO solubilizing capacity to satisfy even the minimum three-fold sink criterion. In contrast, all receptor media containing either 60 or 70% ethanol, as well as physiological saline containing 30% ethanol, fulfilled the minimum sink requirement (Table 10). Among these media, physiological saline containing 60 or 70% ethanol also satisfied the more conservative ten-fold sink criterion (Table 10), thereby providing a larger safety margin for maintaining sink conditions throughout the diffusion experiments.

Based on these results, normal saline containing 60% (*v*/*v*) ethanol was selected as the receptor medium for the in vitro release study through the synthetic polyethersulfone membrane. For the in vitro permeation study, normal saline containing 30% (*v*/*v*) ethanol was selected as the receptor medium.

#### 3.8.2. In Vitro Cinnamon Bark Essential Oil Release Studies

**Figure 12:**
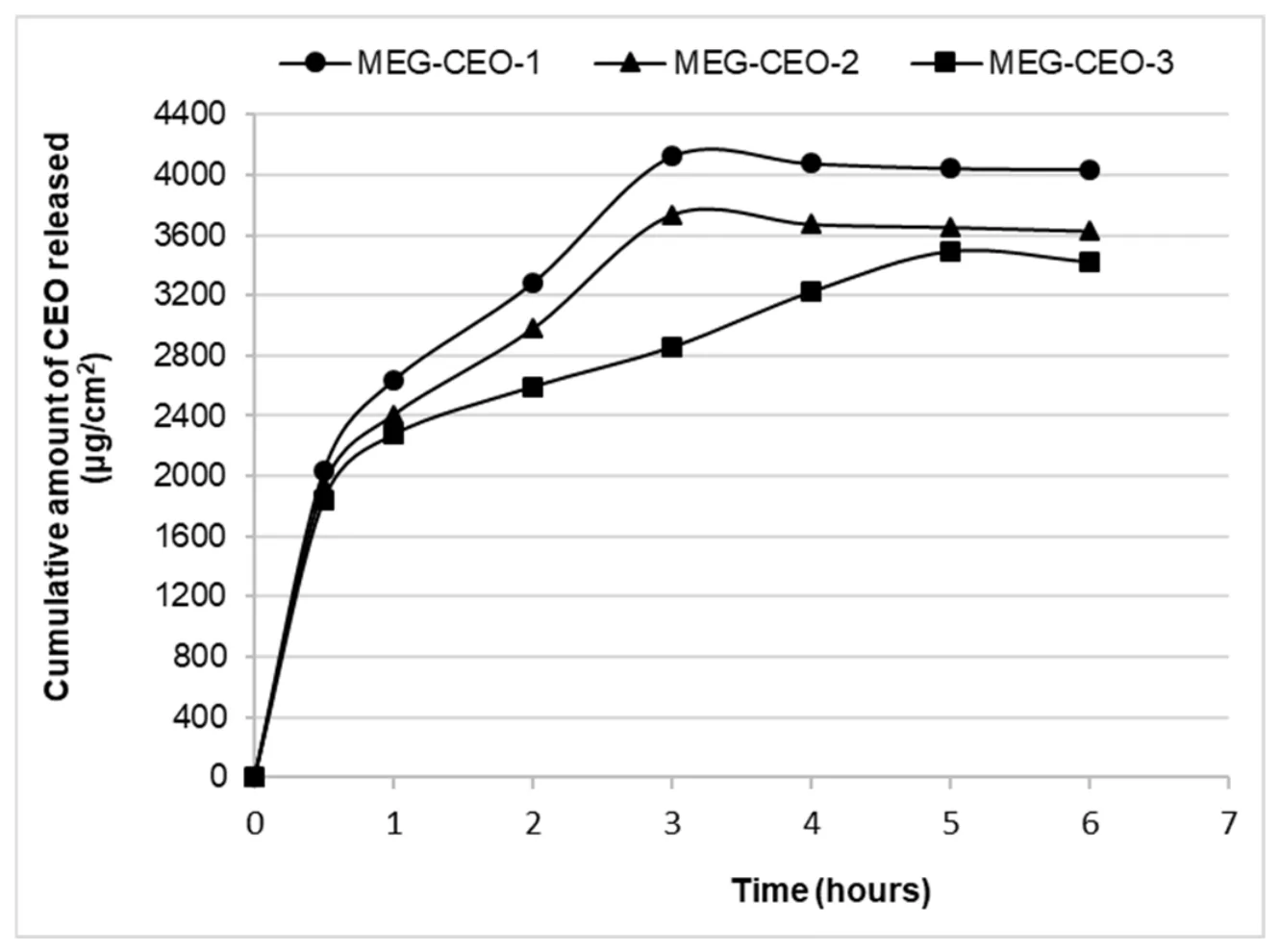
Cumulative amount of CEO released per unit membrane area from the gelled microemulsions during the in vitro release test (IVRT) using Franz diffusion cells equipped with a hydrophilic polyethersulfone membrane (mean ± SD, *n* = 3).

Figure 12 illustrates the cumulative amount of CEO released from the three Aerosil^®^ 200-gelled microemulsions through the polyethersulfone membrane, while the results of mathematical modeling of the obtained release profiles are summarized in Table 11.

Significant differences in release performance were observed among the three formulations. MEG-CEO-1 exhibited the highest release rate constant (1970.80 ± 6.94 μg·cm^−2^·h^−1/2^) and maximum cumulative amount released (4127.16 ± 2.81 μg/cm^2^), followed by MEG-CEO-2 (1705.90 ± 4.90 μg·cm^−2^·h^−1/2^ and 3737.53 ± 5.81 μg/cm^2^, respectively), whereas MEG-CEO-3 showed the lowest release rate constant (1029.80 ± 7.8 μg·cm^−2^·h^−1/2^) and maximum cumulative amounts released (3495.53 ± 6.21 μg/cm^2^). One-way ANOVA followed by Tukey’s post hoc test confirmed significant differences between all three formulations for both parameters (*p* < 0.001). The release rate constant of MEG-CEO-1 was approximately 15% higher than that of MEG-CEO-2 and approximately 1.9-fold higher than that of MEG-CEO-3. The release plateau was reached after approximately 3 h for MEG-CEO-1 and MEG-CEO-2, whereas MEG-CEO-3 continued releasing CEO until approximately 5 h, consistent with greater retention of the essential oil within the more structured formulation matrix.

Among the evaluated equations, the Korsmeyer–Peppas model provided the closest mathematical description of the release profiles, with R2 values ranging from 0.9889 to 0.9999 (Table 11), while the Higuchi model also showed good agreement with the experimental data (R2 = 0.8882–0.9819). The release exponent (n) obtained from the Korsmeyer–Peppas model ranged from 0.239 to 0.344 (Table 11).

#### 3.8.3. In Vitro Skin Permeation Studies

**Figure 13:**
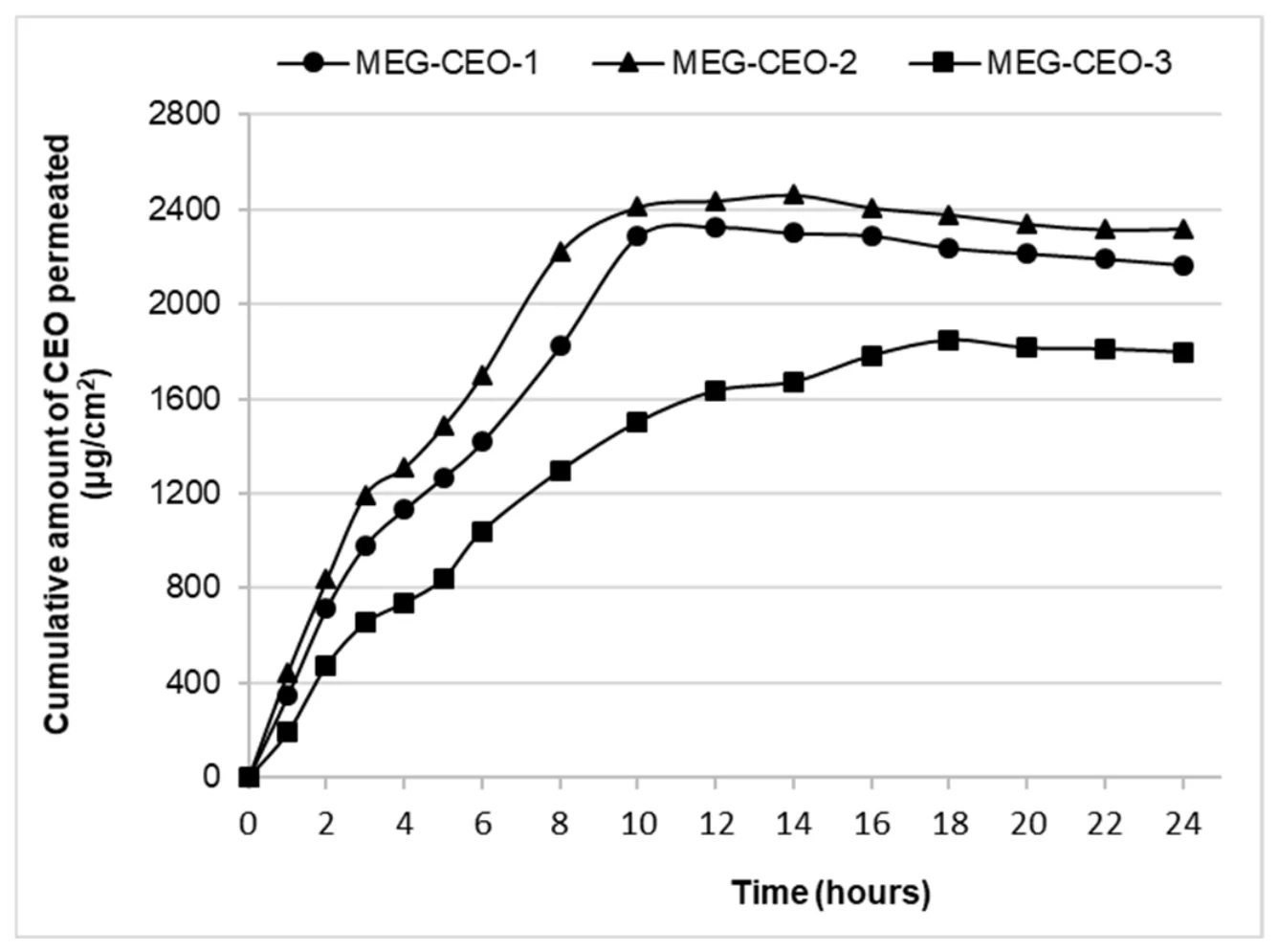
In vitro permeation profiles of CEO from the Aerosil^®^ 200-gelled microemulsions through excised porcine ear skin using Franz diffusion cells. Results are expressed as the cumulative amount of CEO permeated per unit skin area (mean ± SD, *n* = 3).

The cumulative permeation profiles of CEO through excised porcine ear skin are presented in Figure 13.

All formulations exhibited a biphasic permeation behavior characterized by a relatively rapid increase in the permeated amount during the initial hours, followed by a gradual reduction in permeation rate until an apparent plateau was reached (Figure 13). Although MEG-CEO-1 exhibited the highest release rate through the synthetic membrane, MEG-CEO-2 produced the greatest cumulative permeation through porcine skin, whereas MEG-CEO-3 consistently showed the lowest permeation throughout the experiment (Figure 13). This finding indicates that the formulation providing the fastest release is not necessarily the one exhibiting the highest skin permeation, emphasizing the decisive role of the skin barrier in controlling drug transport.

The calculated permeation parameters (Table 12) confirmed the differences observed in the permeation profiles.

Significant differences in skin permeation performance were observed among the three gelled formulations. MEG-CEO-2 exhibited the highest steady-state flux (J_ss_ = 214.17 ± 1.13 µg/cm^2^/h), permeability coefficient (K_P_ = 71.39 ± 0.84 x 10^−6^ cm/h), and permeation rate constant (k_H_ = 913.81 ± µg/cm^2^/h^1/2^), followed by MEG-CEO-1, whereas MEG-CEO-3 showed the lowest values for all three parameters (Table 12). One-way ANOVA followed by Tukey’s post hoc test confirmed significant differences between all three formulations for J_ss_, K_P_, and k_H_ (*p* < 0.05). The superior permeation observed for MEG-CEO-2 most likely reflects an optimal balance between CEO release from the vehicle and its partitioning into the stratum corneum. In contrast, although MEG-CEO-1 released CEO more rapidly through the synthetic membrane, the slightly lower skin permeation suggests that rapid release alone does not necessarily maximize transdermal transport. Conversely, the stronger structural organization of MEG-CEO-3 limited both CEO release and subsequent skin permeation.

Mathematical modeling of the IVPT profiles showed that both the Korsmeyer–Peppas and Higuchi models provided comparatively good descriptions of CEO permeation, although the relative goodness of fit varied among formulations (Table 13).

The Korsmeyer–Peppas model yielded R^2^ values of 0.8783–0.9405, whereas the Higuchi model yielded R^2^ values of 0.8316–0.9524. The Korsmeyer–Peppas equation provided the higher R^2^ values for MEG-CEO-1 and MEG-CEO-2, while the Higuchi model showed the highest R^2^ value for MEG-CEO-3. The permeation exponent (*n*) obtained from the Korsmeyer–Peppas model ranged from 0.492 to 0.664.

## 4. Discussion

The selection of the oil phase was guided by the specific physicochemical characteristics of essential oil, which may represent formulation challenges because of its hydrophobic nature, complex chemical composition and susceptibility of some bioactive constituents to degradation [29,30]. The compositional profile reported in the certificate of analysis of the CEO batch used in this study confirmed cinnamaldehyde as the predominant constituent (75.911%), together with lower proportions of linalool (5.505%) and eugenol (4.297%). Nevertheless, as with other essential oils, the quantitative composition of cinnamon bark essential oil may vary according to botanical and geographical origin, environmental conditions, harvesting and processing factors, extraction method and production batch. Such compositional variability, particularly changes in the relative abundance of cinnamaldehyde and minor constituents, could influence the physicochemical properties, biological activity, and formulation behavior of CEO and should therefore be considered when extrapolating the present findings to oils from different sources or batches. In particular, cinnamaldehyde, the major component of cinnamon bark essential oil, is susceptible to oxidation and other degradation reactions, while the volatile nature of essential oil may adversely affect the long-term stability of microemulsions, which are colloidal delivery systems [29,31]. To improve the physicochemical stability of the system while maintaining a therapeutically relevant CEO concentration (3% *w*/*w*), the oil phase was designed as a binary mixture of CEO and IPM at a mass ratio of 2:1. IPM was selected as a co-oil because of its favorable solvent properties for lipophilic compounds, compatibility with nonionic surfactants, and extensive use in dermal microemulsion formulations [32,33,34,35,36,37]. In addition to facilitating microemulsion formation by improving the properties of the lipophilic phase and interfacial flexibility, IPM may contribute to dermal delivery by enhancing partitioning and diffusion of lipophilic compounds through the stratum corneum [38,39]. Incorporation of IPM into the oil phase was also expected to reduce the viscosity of the lipophilic phase and improve interfacial flexibility, and is thereby expected to facilitate the spontaneous formation of fine microemulsion droplets and enlarge the microemulsion domain within the pseudo-ternary phase diagram [33,34]. In addition to its formulation-related advantages, IPM has been widely reported to enhance the partitioning and diffusion of lipophilic compounds across the stratum corneum, making it particularly suitable for dermal delivery systems. Consequently, the combination of CEO with IPM was expected not only to facilitate microemulsion formation but also to contribute to the dermal delivery performance of the developed formulations. The selected CEO-IPM ratio represented a compromise between formulation requirements and therapeutic considerations. While increasing the proportion of IPM generally facilitates microemulsion formation, excessive dilution of the essential oil would reduce the concentration of the active phytocomplex available for release and skin permeation. Conversely, increasing the CEO content tends to narrow the microemulsion region because of the complex chemical composition of the essential oil. Therefore, a CEO-IPM mass ratio of 2:1 was considered appropriate for simultaneously ensuring formulation stability and maintaining a therapeutically relevant CEO concentration.

Based on the rationale presented in the “Introduction”, decyl glucoside was selected as the surfactant for the development of CEO-loaded cutaneous microemulsions. Subsequently, two pharmaceutically accepted cosurfactants, propylene glycol (PG) and diethyleneglycol monoethyl ether (DEGEE), were selected because both compounds are widely employed in topical microemulsion formulations as multifunctional pharmaceutical excipients combining cosurfactant, cosolvent, and penetration-enhancing properties.

Propylene glycol is one of the most frequently used cosurfactants in dermal microemulsions due to its excellent miscibility with aqueous and surfactant phases. By penetrating the surfactant film, PG reduces interfacial rigidity, enhances film flexibility, and promotes the spontaneous formation of thermodynamically stable microemulsions [7,33,40,41]. In addition, propylene glycol acts as a humectant and a mild skin penetration enhancer, contributing to improved topical performance of the formulation [42,43]. Diethyleneglycol monoethyl ether was selected as an alternative cosurfactant because of its higher solubilization capacity for lipophilic compounds and essential oil constituents, as well as its reported penetration-enhancing properties [30,44]. DEGEE is recognized as one of the most effective pharmaceutical penetration enhancers, promoting drug partitioning into the stratum corneum without causing significant skin irritation [45,46]. Because PG and DEGEE differ in polarity (dielectric constant value of 32.1 and 14.1, respectively) and solvent characteristics [45] both cosurfactants were comparatively investigated to evaluate their influence on the extent of the microemulsion region and the physicochemical properties of the obtained formulations.

In the PG-containing systems, the microemulsion domain increased with increasing DG:PG ratio from 1:1 to 1.5:1, reaching the maximum area at 1.5:1 (770.96 units^2^), followed by a slight decrease at 2:1 (729.22 units^2^). This non-linear response suggests that an intermediate balance between DG and PG was more favorable than either an equimass composition or a higher surfactant proportion. Insufficient surfactant at the 1:1 ratio may have limited interfacial coverage, whereas the lower PG fraction at 2:1 may have reduced the ability of the interfacial film to accommodate the curvature required over a broad range of oil and water contents [47]. A different pattern was observed for DEGEE. The largest microemulsion domain was obtained at the DG:DEGEE ratio of 1:1 (964.70 units^2^), followed by a moderate decrease at 1.5:1 (920.98 units^2^) and a slight increase at 2:1 (932.94 units^2^). Nevertheless, the variation among the three DEGEE-containing systems was relatively limited compared with the overall difference between the two cosurfactants. This finding suggests that DEGEE retained a high capacity to support microemulsion formation across a comparatively broad range of DG:DEGEE ratios. The superior phase-forming performance of DEGEE may be associated with its amphiphilic solvent properties and its ability to partition between the aqueous, oily, and interfacial regions of the system. Incorporation of a cosurfactant into the surfactant film can reduce interfacial rigidity and facilitate the curvature changes required for the formation of isotropic microemulsion structures [48,49,50]. The greater microemulsion areas obtained with DEGEE indicate that, in the present CEO/IPM-DG-water system, it was more effective than PG in expanding the range of compositions capable of forming a single transparent phase. However, the area of the microemulsion domain was not considered the sole criterion for selecting the cosurfactant for subsequent formulation development. The objective of the present study was to develop dermal microemulsions containing CEO with suitable physicochemical characteristics and potential for long-term stability, rather than to maximize the extent of the isotropic phase domain. Particular attention was paid to the chemical stability of cinnamon bark essential oil, whose major constituent, cinnamaldehyde [2,18], is susceptible to oxidative and light-induced degradation [31]. Given the established use of PG in topical formulations and the lack of formulation-specific evidence regarding the long-term chemical compatibility of cinnamaldehyde-rich essential oils with DEGEE-containing decyl glucoside systems, PG was selected as the more conservative cosurfactant for subsequent formulation development. The present findings should also be considered in the context of previous APG-based dermal microemulsion studies. Earlier investigations demonstrated that APG systems can successfully incorporate and deliver chemically diverse active compounds, including ascorbic acid, tacrolimus, lycopene, and adapalene, and that both surfactant structure and formulation composition strongly influence microstructure and skin delivery performance [10,13,14,15,16]. In particular, decyl glucoside/propylene glycol-based systems have previously been shown to provide enhanced dermal delivery of adapalene despite relatively low in vitro release, illustrating that release and skin penetration are not necessarily governed by identical formulation factors [16]. Similarly, APG-based microemulsions containing tea tree oil demonstrated that modification of the APG/cosurfactant ratio substantially altered the microemulsion region and formulation stability [17]. The present study extends these observations by showing that, in a gelled APG-based system containing a complex essential oil cargo, modification of the decyl glucoside/propylene glycol ratio affects not only microemulsion formation but also the mechanical organization of the silica-gelled matrix and its subsequent release and skin-permeation performance. Thus, the relevance of the system lies in establishing a composition-structure-performance relationship across several formulation levels, from phase behavior to semisolid mechanics and biopharmaceutical performance.

Particular attention was paid to the chemical stability of CEO, whose major constituent, cinnamaldehyde [2], is susceptible to oxidation, light-induced degradation, and reactions promoted by alkaline conditions [31]. Since information regarding the long-term chemical compatibility of cinnamaldehyde-rich essential oils with DEGEE-containing decyl glucoside systems remains very limited, propylene glycol was selected as the more conservative cosurfactant for subsequent formulation studies.

The concentration of CEO in the final microemulsion formulations selected from the pseudoternary phase diagrams constructed for the CEO/IPM-DG/PG-water systems was fixed at 3% (*w*/*w*). This concentration represents a compromise between maintaining a therapeutically relevant amount of the active ingredient and preserving the physicochemical stability and dermal tolerability of the formulations. CEO has been incorporated into topical formulations over a relatively broad concentration range; however, concentrations around 2–5% are among the most frequently investigated, because they provide biological activity while minimizing excessive volatility, intense odor, and the potential for skin irritation [51,52]. Since the CEO:IPM ratio was maintained at 2:1 (*w*/*w*), the selected CEO concentration resulted in a total oil phase content of 4.5% (*w*/*w*). Maintaining the oil phase at a relatively low concentration also facilitates spontaneous microemulsion formation and allows the preparation of oil-in-water (O/W) systems with a high aqueous content, desirable for topical administration because of their non-greasy sensory characteristics. Within each pseudo-ternary phase diagram, formulations containing 45% (*w*/*w*) S_mix_ were selected because this composition was located close to the center of the isotropic microemulsion domain, thereby minimizing the risk of phase separation associated with minor compositional variations during preparation and storage, while avoiding unnecessarily high surfactant concentrations [17].

The determined characteristics of selected O/W CEO-loaded microemulsions, including droplet size and size distribution, zeta potential, dynamic viscosity, refractive index, and apparent pH together, provide complementary information on their colloidal organization, homogeneity, flow properties, optical characteristics, and suitability for dermal application [53]. The nanometric droplet size obtained for all formulations is characteristic of microemulsion systems and agrees with their transparent and optically homogeneous appearance observed during visual examination. Similar droplet size ranges have been reported for alkyl polyglucoside (APG)-based topical microemulsions including curcumin-loaded O/W systems (45–81 nm) and physically stable APG/propylene glycol microemulsions containing tea tree oil with nanometric aggregates [17,29]. A progressive decrease in mean droplet diameter was observed from ME-CEO-1 to ME-CEO-3, concomitant with the increase in decyl glucoside concentration from 30% (*w*/*w*) to 36.0% (*w*/*w*) and the corresponding decrease in propylene glycol content. ME-CEO-3, containing the highest surfactant-to-cosurfactant ratio, exhibited the smallest droplets (24.95 ± 0.35), whereas ME-CEO-1 showed the largest mean diameter (34.62 ± 0.18) (Table 2). This trend may reflect more efficient interfacial coverage at higher DG concentration, resulting in a reduction in the specific dimensions of the dispersed oil phase. However, because surfactant and cosurfactant contents varied simultaneously, the observed effect should be attributed to the overall S_mix_ composition rather than exclusively to DG concentration [41]. The dependence of APG-based microemulsion structure and solubilization behavior on surfactant composition and HLB has also been demonstrated in previously studied microemulsion systems [10]. The PDI values ranged from 0.136 ± 0.023 to 0.312 ± 0.016 (Table 2). ME-CEO-1 and ME-CEO-3 exhibited narrow droplet size distributions, with PDI values of 0.144 ± 0.024 and 0.136 ± 0.023, respectively, indicating highly uniform colloidal populations. ME-CEO-2 presented a broader, although still acceptable, distribution (PDI = 0.312 ± 0.016), suggesting greater structural heterogeneity at an intermediate DG-PG ratio. The fact that the intermediate formulation showed the highest PDI value, whereas ME-CEO-3 combined the smallest droplet diameter with the lowest PDI, indicates that the relationship between S_mix_ composition and droplet organization was not strictly linear. Similar composition-dependent changes in aggregate size and distribution have been reported for APG-containing systems, in which changes in water and surfactant contents were associated with transitions among W/O bicontinuous and O/W structure [17,47].

All tested formulations exhibited slightly negative, near-neutral zeta potential values, ranging from −0.927 ± 0.24 to −0.726 ± 0.28 mV (Table 2), with no statistically significant differences among the investigated compositions (*p* > 0.05). These values are consistent with the nonionic nature of decyl glucoside and indicate that electrostatic repulsion is unlikely to represent the principal stabilization mechanism in these systems. Instead, stabilization is expected to arise predominantly from the interfacial organization of DG and PG and from steric and hydration effects associated with the glucoside head groups. Consequently, zeta potential has limited predictive value for the colloidal stability of these nonionic microemulsions, and the near-zero values should not be interpreted according to conventional zeta-potential thresholds commonly applied to electrostatically stabilized dispersions. In the present systems, zeta potential should therefore be regarded primarily as a complementary electrokinetic characterization parameter rather than as an independent predictor of long-term colloidal stability. Near-zero zeta potential values have also been reported for other cutaneous microemulsions stabilized with nonionic surfactants [10,54], supporting the concept that low electrokinetic potential does not preclude the formation of physically homogeneous nonionic microemulsion systems.

The investigated microemulsion systems exhibited dynamic viscosity values, ranging from 26 ± 1.82 to 28 ± 3.27 mPa·s (Table 2). The narrow range and overlapping variability indicate that modification of the DG:PG ratio within the investigated interval had no pronounced effect on the bulk viscosity of the systems. These findings are consistent with the readily flowing nature of the formulations and with the generally low viscosity of unstructured O/W microemulsions, including APG-based systems [29]. However, the low viscosity of the parent microemulsions may limit their residence time after topical application, supporting the need for subsequent gelation to improve application properties and formulation retention on the skin [17,47].

It should be noted that, because all the investigated formulations (microemulsions and gelled microemulsions) are multicomponent colloidal systems rather than simple aqueous solutions, the potentiometrically determined values are more appropriately regarded as apparent pH values and should not be interpreted as rigorous thermodynamic pH measurements. The statistically significant differences observed among the apparent pH values of the three CEO-loaded microemulsions do not necessarily imply practical or formulation-related relevance, since all systems remain within the same mildly acidic range.

The spectrophotometric assay confirmed excellent retention of CEO during gelation, with measured contents ranging from 99.41 ± 0.81% to 100.78 ± 0.43% of the theoretical value, with deviations of less than 1% from the nominal concentration. The obtained results indicate negligible losses of the volatile active ingredient during gelation and demonstrate the accuracy and reproducibility of the preparation procedure. No systematic differences in CEO content were observed among the three formulations despite their different DG:PG ratios, suggesting that the composition of the surfactant-cosurfactant mixture had no detectable influence on CEO incorporation into the gelled systems. The very small deviations from the nominal concentration are within the expected analytical variability and demonstrate the homogeneous distribution of the essential oil throughout the formulations [17,29].

The apparent pH values determined for the gelled microemulsions (Table 3) were within the target interval established during formulation development and are considered appropriate for topical administration [55,56,57]. Maintaining the formulations within this apparent pH range is expected to improve dermal compatibility while avoiding strongly alkaline conditions that may accelerate degradation of cinnamaldehyde, the principal constituent of CEO [58]. The minor differences among formulations likely resulted from the individual apparent pH adjustment procedure rather than the DG:PG ratio. Although these differences were statistically significant, their relatively small magnitude is unlikely to imply major differences in dermal compatibility, as all formulations remained within a mildly acidic to near-neutral apparent pH range. This finding confirms that the apparent pH adjustment procedure was robust and reproducible over the investigated formulation range.

All gelled formulations exhibited non-Newtonian shear-thinning behavior, characterized by a progressive decrease in apparent viscosity with increasing shear rate. Furthermore, a distinct hysteresis loop between the ascending and descending flow curves was observed for all systems, demonstrating pronounced time-dependent thixotropic behavior [59,60]. The observed rheological characteristics are typical of semisolid systems structured with hydrophilic fumed silica. Aerosil^®^200 forms a three-dimensional network through hydrogen bonding between silanol groups located on the particle surface. At rest, this interconnected structure confers high structural integrity and resistance to deformation. Upon increasing shear, progressive disruption of the silica network results in a marked reduction in viscosity, whereas partial rebuilding of the structure occurs once the applied stress is removed, giving rise to the hysteresis loop characteristic of thixotropic materials [61,62,63]. For each DG:PG ratio, incorporation of CEO resulted in a consistent increase in apparent viscosity compared with the corresponding blank gelled microemulsion. The increase was particularly evident for MEG-CEO-2 and MEG-CEO-3, suggesting that the dispersed oil phase contributed to reinforcement of the internal microstructure generated by Aerosil^®^200. This behavior may be attributed to interactions between the silica network and the oil-rich microemulsion droplets, which act as additional structural elements within the three-dimensional gel matrix.

A similar trend was observed for thixotropy, with the hysteresis area increasing from 46 to 49% in blank formulations to 59–65% after CEO incorporation. According to the adopted criterion (AR > 5%), all formulations exhibited marked thixotropic behavior. The larger hysteresis areas after CEO incorporation indicate enhanced reversible structural breakdown and recovery under shear. Notably, the highest thixotropy was observed for MEG-CEO-2 rather than the most viscous formulation (MEG-CEO-3), suggesting that structural recovery depends on gel network organization in addition to viscosity. Due to pronounced thixotropic behavior of the formulations, mathematical modelling of the steady-shear flow curves was not pursued. Although several constitutive equations have been proposed to describe time-dependent non-Newtonian materials, no universally accepted rheological model adequately represents the complex structural breakdown and rebuilding phenomena occurring in thixotropic systems [59,60].

As recently emphasized by Cook [64], numerous phenomenological models have been developed for specific classes of complex fluids, each involving adjustable parameters that are often system-dependent and lack universal physical significance. Therefore, the rheological characterization of the present formulations was based on the experimental flow curves, apparent viscosity profiles, and thixotropy index, which provide a direct and reliable description of their flow behavior. Moreover, preliminary attempts to fit the experimental data using generalized viscosity models resulted in poorly identifiable fitting parameters despite high coefficients of determination, indicating that the estimated parameters had limited physical meaning within the investigated shear-rate range. Consequently, only the experimental rheological data are presented and discussed. From a pharmaceutical perspective, this rheological behavior (combination of pronounced shear-thinning behavior and marked thixotropy) is particularly advantageous for topical administration. The relatively high viscosity at rest contributes to formulation stability and prolonged residence at the application site, whereas the pronounced shear-thinning behavior facilitates spreading under the mechanical stress generated during application. After removal of the applied force, partial recovery of the internal structure is expected to minimize formulation runoff from the skin surface [65].

All investigated gel formulations exhibited a well-defined linear viscoelastic region, characterized by nearly constant values of G’ and G” over the low-stress domain, indicating that the internal gel structure remained essentially undisturbed under small oscillatory deformations. A comparable linear viscoelastic response has been reported for hydrophilic fumed silica gels and suspensions, where an extended LVR reflects the presence of a stable particle network capable of withstanding small-amplitude deformations without structural disruption. Although those studies generally involved simpler continuous media than the APG-based microemulsions investigated here, the overall rheological behavior was qualitatively similar [59,66]. Non-linear viscoelastic behavior of fumed silica suspension. The existence of a well-defined LVR confirms that the oscillatory measurements performed subsequently (frequency sweep) were conducted under non-destructive conditions, ensuring that the measured viscoelastic parameters reflected the intrinsic structure of the formulations rather than deformation-induced structural changes.

For all formulations, the storage modulus was markedly higher than the loss modulus throughout the LVR, with G’ values ranging from approximately 170 to 463 Pa, whereas G” varied between 8 and 27 Pa. The predominance of the elastic component demonstrates that the investigated systems behaved as solid-like viscoelastic materials, which is characteristic of colloidal gel networks formed by hydrophilic fumed silica. The predominance of the elastic modulus over the viscous modulus agrees with previous observations for Aerosil^®^-structured colloidal gels, in which hydrogen-bond-mediated particle aggregation gives rise to a percolated network exhibiting solid-like mechanical behavior [67]. Incorporation of CEO consistently increased the elastic modulus of the systems. For each DG:PG ratio, the corresponding CEO-loaded gel formulation exhibited higher G’ values (by approximately 1.2-fold for MEG-CEO-1, 2.0-fold for MEG-CEO-2 and 2.2-fold for MEG-CEO-3) than the respective blank gel, the effect being more pronounced with increasing surfactant content. These findings indicate that the dispersed CEO-containing microemulsion droplets reinforce the silica network, resulting in a stiffer and mechanically stronger gel structure. Although direct comparisons with the literature data are difficult because APG-based microemulsion gels have been scarcely investigated, similar increases in the elastic modulus after strengthening of the internal particulate network have been described for fumed silica-based gel systems [67]. A similar but less pronounced trend was observed for the loss modulus, indicating that incorporation of CEO also increased the viscous component of the systems. Nevertheless, the increase in G” was considerably greater than that of G’, suggesting that the essential oil preferentially strengthened the elastic component of the three-dimensional network rather than simply increasing viscous resistance. The loss factor (tan δ), defined as the ratio of G”/G’, remained well below unity for all formulations (0.042–0.065), confirming the predominance of elastic over viscous behavior. Such tan δ values are generally associated with highly structured gel systems possessing a stable interconnected network capable of resisting deformation under low applied stresses. Comparable loss-factor values (tan δ << 1) have been reported for highly structured silica gels, confirming that the elastic response predominates over viscous dissipation in these systems [60]. Although slight variations in tan δ values were observed after CEO incorporation, all formulations remained within a narrow range, indicating that the overall viscoelastic balance of the systems was preserved. Therefore, incorporation of the essential oil mainly reinforced the gel structure without fundamentally altering the relative contributions of the elastic and viscous components.

For all formulations, G’ remained consistently higher than G” throughout almost the entire investigated frequency range, confirming that the elastic component predominated over the viscous response under non-destructive oscillatory conditions. Both G’ and G” exhibited only limited frequency dependence over the low-and intermediate frequency domains, particularly in the case of CEO-loaded formulations. The relatively constant values of the storage modulus indicate the presence of a mechanically stable three-dimensional network whose elastic response was only weakly affected by the deformation timescale. Such behavior is generally considered characteristic of well-developed colloidal gel structures [66,67].

Compared with the corresponding blank formulations, incorporation of CEO resulted in systematically higher storage modulus values over the entire frequency range, whereas the overall shape of the frequency spectra remained essentially unchanged. These findings suggest that CEO strengthened the existing silica network without altering the fundamental viscoelastic nature of the gel structure. Although direct comparison with the literature is limited by the complexity of the present APG-based microemulsion systems, the effect of CEO incorporation on G’ is qualitatively consistent with previous reports of Sugino et al. [67]. The complex viscosity decreased progressively with increasing oscillation frequency for all investigated systems, reflecting the reduced resistance to deformation under rapidly oscillating conditions; this behavior agrees with the dynamic behavior generally described for physically crosslinked colloidal gels, whose internal network progressively adapts to faster deformation rates [60]. This behavior is consistent with the shear-thinning characteristics previously observed during steady-shear measurements and confirms the structural rearrangement capability of the silica network under dynamic loading. The CEO-loaded formulations showed higher complex viscosity values than their corresponding blank systems throughout the investigated frequency domain, further confirming that incorporation of the essential oil contributed to reinforcement of the internal gel network. From a pharmaceutical perspective, the predominance of the elastic response together with the limited frequency dependence of the storage modulus indicates that the investigated formulations possess a sufficiently stable internal structure to withstand the relatively low-frequency mechanical stresses encountered during storage and topical administration, while still allowing structural adaptation during application to the skin.

Yield stress represents the critical stress required to initiate irreversible structural breakdown of the gel network and therefore provides a direct measure of the mechanical strength of the formulations [68]. Oscillatory amplitude sweep measurements are particularly suitable for structured colloidal gels, as they determine the onset of structural yielding under controlled deformations [69]. Compared with steady-shear extrapolation methods, they minimize the effects of extrapolation and wall slip, providing a more reliable assessment of yield stress and network disruption [70]. For all DG:PG ratios, CEO incorporation increased the yield stress of the gelled systems, from 3.450 ± 2.38–5.374 ± 1.62 Pa in the blank formulations to 6.401 ± 3.18–9.030 ± 2.55 Pa in the corresponding CEO-loaded systems (Table 5), indicating reinforcement of the Aerosil^®^ 200 gel network and greater resistance to structural deformation. The yield stress results were consistent with the oscillatory stress sweep measurements. The lack of a strictly proportional relationship between storage modulus and yield stress suggests that yielding depends on both network stiffness and the structural organization of the silica gel network. From a practical standpoint, higher yield stress values are advantageous for topical semisolid formulations because they improve stability during storage and reduce the tendency of the formulation to flow under its own weight after application. At the same time, the measured yield stresses remained sufficiently low to allow spreading on the skin under the relatively small stresses generated during manual application, indicating an appropriate balance between formulation stability and ease of use [60].

The consistency of the gelled formulations was evaluated using two complementary methods, namely penetrometry and parallel-plate spreadability test (Figure 9). Whereas the penetration test provided information on formulation hardness under localized compression, the spreadability test evaluated the deformation behavior of the gels under progressively increasing compressive loads. Together, these measurements provide a practical assessment of the mechanical performance of topical semisolid formulations during handling and skin application [71]. The penetrometric results are in excellent agreement with the rheological characterization. Formulations exhibiting higher storage modulus (G’), higher apparent viscosity and higher yield stress also showed lower penetration values, confirming that increased viscoelastic rigidity translated into greater resistance to localized mechanical deformation. Similar relationships between penetration resistance and oscillatory rheological parameters have been reported for semisolid pharmaceutical systems, where increased gel strength is generally associated with higher elastic modulus and greater structural integrity. Consequently, penetrometry is widely regarded as a simple complementary technique for assessing the mechanical consistency of topical formulations [25]. For all formulations, the spreading area increased progressively with increasing compressive load (Figure 9), indicating continuous deformation of the gel structure under externally applied stress. Although in the presence of CEO the formulation hardness increased, the corresponding formulations generally produced larger spreading areas than the blank gels under identical loads. This observation suggests that, once the yield point had been exceeded, the CEO-loaded systems were capable of undergoing controlled plastic deformation while maintaining structural cohesion.

The spreadability profiles are consistent with the pronounced shear-thinning behavior observed during steady-shear tests. Under increasing compressive stress, disruption and rearrangement of the silica network reduced internal resistance to deformation, allowing the gels to spread efficiently despite their relatively high structural rigidity at rest. This combination of high structural stability during storage and good spreadability during application represents a desirable characteristic for topical dermal formulations [71,72]. Similar behavior has been described for silica-thickened semisolid formulations and topical gels, where increased elastic modulus at rest coexists with satisfactory spreadability owing to the reversible breakdown of the three-dimensional network during application. Such behavior is considered advantageous for dermal dosage forms because it facilitates administration while minimizing undesired flow during storage.

Overall, the consistency measurements corroborated the rheological findings, demonstrating that incorporation of CEO produced mechanically stronger gel networks while preserving excellent deformability under externally applied stress.

Firmness increased consistently after incorporation of CEO, suggesting greater resistance of the formulations to compressive deformation. Compared with the corresponding blank formulations, firmness increased by approximately 2.0-fold, 2.5-fold and 1.9-fold for MEG-CEO-1, MEG-CEO-2 and MEG-CEO-3, respectively. Among all investigated systems, MEG-CEO-3 exhibited the highest firmness, reflecting the formation of the mechanically strongest gel structure. A similar trend was observed for consistency, which increased following incorporation of CEO in all formulations. Compared with the corresponding blank gels, consistency increased by approximately 1.6-fold, 2.4-fold and 1.8-fold for MEG-CEO-1, MEG-CEO-2 and MEG-CEO-3, respectively. Since consistency reflects the work required to deform the material during compression [73], these findings further demonstrate that CEO loading enhanced the mechanical strength and structural integrity of the three-dimensional Aerosil^®^ 200 gel network. Comparable increases in firmness and consistency have been reported for semisolid formulations exhibiting high elastic modulus and strong internal gel networks, confirming the close relationship between textural and rheological properties of pharmaceutical gels [62,74,75]. The absolute values of cohesiveness and viscosity index increased even more markedly following CEO incorporation. Compared with the corresponding blank formulations, both parameters increased by approximately 2.2–2.6-fold for MEG-CEO-1 and MEG-CEO-2, and by 3.4–3.7-fold for MEG-CEO-3. These pronounced increases indicate greater internal cohesion and enhanced resistance of the gel structure during the probe withdrawal [73]. Despite the increased firmness and work of shear, all formulations exhibited smooth and continuous spreading profiles without abrupt force fluctuations, suggesting gradual deformation rather than brittle structural failure. This behavior reflects the reversible disruption of the silica network during deformation, which is characteristic of physically structured semisolid systems [76]. Similar texture profiles have been described for physically cross-linked topical gels, in which high mechanical strength at rest is combined with progressive structural breakdown during spreading, facilitating dermal application while maintaining adequate formulation stability [62,75]. Overall, texture analysis corroborated the rheological and consistency measurements, demonstrating that incorporation of CEO produced mechanically stronger gel networks while preserving controlled deformability during compression and spreading. The convergence of these independent characterization techniques provides strong evidence that CEO reinforced the Aerosil^®^ 200-based three-dimensional structure without compromising the practical applicability of the formulations.

The preliminary short-term stability assessment provided additional evidence regarding the maintenance of physicochemical characteristics of both CEO-loaded microemulsions and gelled microemulsions under the investigated storage conditions. The combined evaluation of droplet size, size distribution, and macroscopic characteristics provides complementary information on the physicochemical behavior of microemulsion systems during storage [53]. The absence of visible macroscopic changes, together with the lack of statistically significant time-dependent variations in the investigated parameters, except for the apparent pH of MEG-CEO-2, supports the short-term physical stability of both formulation types. For the liquid microemulsions, the maintenance of droplet diameter and polydispersity index suggests that no appreciable changes in droplet size and size distribution occurred during the observation period. Although the near-neutral zeta potential values are consistent with the nonionic nature of the surfactant system, they should not be regarded as an independent indicator of colloidal stability, since steric and hydration-related mechanisms may contribute substantially to the stabilization of these systems. The absence of significant variations in dynamic viscosity and apparent pH further supports the maintenance of the physicochemical characteristics of the liquid formulations. For the gelled microemulsions, the maintenance of apparent viscosity and penetration value suggests that their semisolid characteristics were preserved during storage, without evidence of progressive softening or hardening. Furthermore, the absence of significant changes in CEO content is consistent with the retention of the overall essential oil content within the gelled formulations over the investigated period. Collectively, these findings support the maintenance of the principal physicochemical characteristics of both formulation types during 45 days of storage.

A significant overall effect of storage time on apparent pH was identified for MEG-CEO-2; however, subsequent pairwise comparisons with baseline, adjusted using the Holm procedure, revealed no statistically significant differences. Moreover, the observed variations were small and did not follow a progressive trend, with the apparent pH at day 45 remaining close to its initial value. Therefore, although the overall time effect was statistically significant, the magnitude and pattern of the observed variations suggest that this finding has limited practical relevance to the short-term physicochemical stability of the formulation.

The preliminary calculations indicated that a minimum CEO solubility of approximately 0.0041 mL/mL was required to maintain the three-fold sink condition, while a more conservative ten-fold sink criterion would require approximately 0.0135 mL/mL. These values were used to guide the selection of suitable receptor media for further evaluation [77,78,79,80,81]. Based on their solubilizing capacity and compatibility with diffusion studies, six hydroethanolic receptor media were selected for experimental evaluation. Phosphate-buffered saline (PBS, pH 7.4) and physiological saline were considered due to their physiological relevance and frequent use in Franz diffusion experiments [79,81,82]. Ethanol was added at increasing concentrations (30, 60, and 70%, *v*/*v*) to enhance CEO solubility while minimizing potential effects on membrane integrity and formulation stability [80,82,83]. Normal saline containing 60% (*v*/*v*) ethanol was selected for the in vitro release study because it combines adequate CEO solubilizing capacity with a simpler composition than PBS (pH 7.4). For the in vitro release study, 60% (*v*/*v*) ethanol in normal saline was selected because it provided sufficient CEO solubilization with a simpler composition than PBS. In contrast, 30% (*v*/*v*) ethanol in normal saline was chosen for the permeation study, as it fulfilled the sink requirement while reducing the risk of ethanol-induced disruption of the skin barrier [82,84].

The in vitro release test (IVRT) is an essential tool for the physicochemical characterization of topical drug delivery systems, enabling evaluation of the intrinsic release performance of the formulation independently of biological barriers [79,83]. In addition, mathematical modeling of the release profiles provides valuable information on the kinetics and underlying mechanism of active ingredient release, facilitating interpretation of the effect of formulation variables on drug release behavior [85,86]. All formulations exhibited a rapid initial release during the first hour, followed by a slower release phase that gradually approached a plateau (Figure 12). Such biphasic behavior is characteristic of diffusion-controlled release systems, in which an initially high concentration gradient promotes rapid partitioning of the active compound into the receptor medium, whereas the release rate progressively decreases as the concentration gradient diminishes. This release pattern has been widely reported for microemulsion-based topical delivery systems containing lipophilic active compounds [9,14,50]. Although all formulations contained the same concentration of CEO, identical oil phase content and the same amount of Aerosil^®^ 200, clear differences in the release profiles were observed. MEG-CEO-1 showed the highest CEO release rate and cumulative amount released, followed by MEG-CEO-2, whereas MEG-CEO-3 exhibited the slowest and most prolonged release (Figure 12). Since the three formulations differ only in the surfactant-to-cosurfactant ratio, the observed differences can reasonably be attributed to changes in the internal organization of the microemulsion droplets. Increasing the decyl glucoside proportion relative to propylene glycol is expected to produce a more rigid interfacial film and stronger interactions between the dispersed oil domains and the surrounding gel matrix [15]. CEO diffusion from the internal oil phase into the external aqueous phase becomes progressively slower, resulting in lower release rates.

The release profiles were also consistent with the rheological and textural properties of the formulations. MEG-CEO-3, which presented the highest structural strength, firmness and consistency, showed the lowest CEO release rate, whereas MEG-CEO-1 combined the weakest gel structure with the fastest release. This inverse relationship suggests that the increasingly resistant silica network reduced CEO molecular mobility by increasing diffusional resistance within the continuous aqueous phase.

Among the evaluated equations, the Korsmeyer–Peppas model provided the closest mathematical description of the IVRT profiles, with R2 values ranging from 0.9889 to 0.9999 (Table 11). The Korsmeyer–Peppas equation is a semi-empirical power-law model and was applied here as a comparative tool for describing CEO release from the structurally complex gelled microemulsions rather than as a definitive mechanistic representation of the system. Its applicability in the present context is supported by the multicomponent nature of CEO transport, which may involve partitioning from the dispersed oil domains, diffusion through the aqueous continuous phase and the Aerosil^®^ 200-structured network, and subsequent transport across the hydrophilic membrane. The Higuchi model also showed good agreement with the experimental data (R2 = 0.8882–0.9819), particularly for MEG-CEO-1 and MEG-CEO-2, supporting an important contribution of diffusion to the overall release process. In contrast, the lower coefficients of determination obtained with the zero-order model indicate that constant-rate release did not adequately describe these systems.

The Korsmeyer–Peppas release exponents were relatively low (n = 0.239–0.344) and should be interpreted cautiously. Conventional threshold values used to classify Fickian, anomalous, and Case-II transport depend on the geometry and physical assumptions of the delivery matrix and were established primarily for systems with well-defined matrix geometries; therefore, their direct application to structurally heterogeneous Aerosil^®^ 200-gelled microemulsions is not straightforward [87]. Accordingly, the low *n* values observed in the present study should not, by themselves, be considered definitive evidence of a specific molecular transport mechanism. Rather, when considered together with the comparatively good Higuchi fits and the structural characteristics of the formulations, they are consistent with a predominantly diffusion-influenced release process occurring within a complex multicomponent system in which several transport steps may contribute to the overall release profile.

In vitro permeation testing (IVPT) using excised porcine skin is a widely accepted approach for evaluating the ability of topical formulations to deliver active ingredients across the skin barrier under controlled experimental conditions [84]. The biphasic permeation behavior observed for the studied gelled microemulsion formulations is commonly reported in topical delivery systems and may reflect the progressive reduction in the concentration gradient across the skin, together with the diffusional resistance and partitioning processes associated with the skin barrier [84,88].

Although MEG-CEO-2 exhibited the highest cumulative permeation and steady-state flux among the investigated formulations under the experimental IVPT conditions, these findings should not be interpreted as direct evidence of superior therapeutic performance. At present, no validated cutaneous concentration or exposure threshold can be used to relate the amount of permeated CEO to a clinically effective dose. Furthermore, for a formulation intended for local dermal application, extensive permeation across the entire skin barrier is not necessarily the desired endpoint, since retention of the active constituents within the skin layers may be more relevant to local therapeutic activity. The present IVPT study therefore provides a comparative assessment of the skin-delivery performance of the formulations rather than evidence of therapeutic efficacy. Further studies addressing CEO retention and distribution within the relevant skin layers, together with appropriate pharmacodynamic and in vivo investigations, are required to establish whether the observed differences in permeation translate into clinically relevant effects.

Comparison of the IVRT and IVPT results further demonstrates that release and skin permeation cannot be considered equivalent processes. Whereas the synthetic membrane primarily evaluates CEO release from the formulation, porcine skin represents a complex biological barrier in which partitioning into the stratum corneum, diffusion across multiple skin layers, and interactions with skin lipids all contribute to the overall permeation process. Consequently, formulation variables that favor rapid release do not necessarily produce the highest skin permeation [82,84].

The kinetic analysis indicates that CEO permeation through excised porcine skin cannot be adequately represented by a single simple transport mechanism across all three formulations. Both Higuchi and Korsmeyer–Peppas equations provided comparatively good fits, with their relative goodness of fit depending on formulation composition. The agreement with the Higuchi model, particularly for MEG-CEO-3 (R2 = 0.9524), supports an important diffusional contribution to CEO transport [89]. The Korsmeyer–Peppas model provides a useful semi-empirical description of the permeation profiles but should not be regarded as a mechanistic model of the skin barrier [90]. Under IVPT conditions, the measured permeation profile represents a composite process involving release from the formulation, partitioning into the stratum corneum, and diffusion through successive skin layers; consequently, the fitted exponent reflects the overall transport behavior rather than a single elementary diffusion mechanism.

The *n* values obtained during IVPT (0.492–0.664) were higher than those observed during IVRT (0.239–0.344). Although this difference is consistent with the greater complexity introduced by the biological membrane, conventional Korsmeyer–Peppas threshold values should not be used to assign a specific transport mechanism to these heterogeneous formulation-skin systems. The Korsmeyer–Peppas parameters are therefore interpreted comparatively and in conjunction with the Higuchi fits, the physicochemical organization of the formulations, and the experimentally determined permeation parameters, rather than as standalone mechanistic descriptors.

### Limitations and Future Perspectives

The present study has several limitations that should be considered when interpreting the findings. First, although the developed gelled microemulsions were extensively characterized from a physicochemical and pharmacotechnical perspective, the stability assessment was limited to a preliminary 45-day investigation under a single storage condition. While the results supported the maintenance of the principal physicochemical characteristics of both the liquid and gelled microemulsions during this period, long-term and accelerated stability was not evaluated. This limitation is particularly relevant for cinnamon bark essential oil because of the volatility and susceptibility to oxidation of its constituents, especially cinnamaldehyde. Furthermore, although the overall CEO content was maintained during storage, the chemical stability of individual essential oil constituents was not specifically investigated. Future studies should therefore extend the stability assessment to longer storage periods and accelerated conditions, with particular emphasis on component-specific chemical analysis, including cinnamaldehyde retention and the identification of potential degradation products. Second, the present work focused on formulation development and biopharmaceutical performance and did not investigate whether incorporation into the gelled microemulsions preserves or modifies the biological activities attributed to CEO. Comparative studies of free and formulated CEO using appropriate antimicrobial, antioxidant, and anti-inflammatory assays are therefore required to establish whether the developed delivery systems maintain or potentially enhance its biological activity. Third, although the IVPT experiments using excised porcine skin provided relevant information on CEO permeation across a biological barrier, these data alone cannot establish therapeutic efficacy or predict the concentrations achieved at specific cutaneous target sites. Further studies should therefore investigate skin retention and distribution of CEO and/or its major constituents within different skin layers and relate these findings to biologically effective concentrations. Finally, the present ex vivo permeation results require confirmation in appropriate biological models, together with evaluation of local tolerability and safety, before conclusions regarding therapeutic efficacy or clinical applicability can be drawn. Collectively, these investigations will be necessary to establish the long-term stability, biological performance, dermal disposition, safety, and therapeutic potential of the developed CEO-loaded gelled microemulsions.

## 5. Conclusions

This study demonstrated the successful development of oil-in-water CEO-loaded microemulsions and their subsequent transformation into Aerosil^®^ 200-gelled semisolid systems for dermal delivery. The microemulsions were rationally selected from the isotropic regions of pseudoternary phase diagrams constructed using different decyl glucoside:propylene glycol ratios and converted into gel formulations while preserving their physicochemical characteristics. The obtained gelled microemulsions exhibited favorable physicochemical, rheological, textural, release, and permeation properties, supporting their further evaluation as potential topical delivery systems for cinnamon bark essential oil.

The results demonstrated that the decyl glucoside:propylene glycol ratio influenced formulation structure and performance, affecting rheological behavior, mechanical properties, and CEO release characteristics. Higher decyl glucoside proportions generally increased gel strength, firmness, consistency, cohesiveness and yield stress while reducing CEO release through the synthetic membrane, indicating that formulation performance can be modulated through compositional optimization without changing the active ingredient content.

The combined IVRT and IVPT studies provided complementary information regarding formulation performance. Whereas the synthetic membrane primarily reflected the intrinsic release behavior of CEO from the gelled microemulsions, permeation through excised porcine ear skin demonstrated that cutaneous delivery is influenced not only by CEO release from the formulation but also by the interaction of the formulation with the biological barrier.

Among the investigated formulations, MEG-CEO-2 provided the most favorable balance among the experimentally evaluated mechanical, release, and skin permeation characteristics, whereas MEG-CEO-3 exhibited the greatest structural strength but the lowest release and permeation rates. Nevertheless, the higher skin permeation observed for MEG-CEO-2 should not be interpreted as evidence of superior therapeutic performance, since cutaneous retention and layer-specific distribution of CEO were not evaluated.

Overall, the findings demonstrate that adjustment of the decyl glucoside:propylene glycol ratio is an effective approach for modulating the pharmacotechnical and biopharmaceutical performance of APG-based gelled microemulsions for dermal delivery. The developed systems combine the solubilization capacity of microemulsions with the improved handling properties of semisolid formulations. The preliminary 45-day stability assessment further supported the maintenance of the principal physicochemical characteristics of both the liquid and gelled microemulsions under the investigated storage conditions. However, the biological activity of CEO following incorporation into the gelled microemulsions was not evaluated in the present study, and preservation of its reported antimicrobial, antioxidant, and anti-inflammatory properties therefore remains to be experimentally established. Further studies should investigate long-term and accelerated formulation stability, including the chemical integrity of individual CEO constituents; compare the biological activity of free and formulated CEO; investigate cutaneous retention and layer-specific distribution; and subsequently evaluate in vivo performance to establish the therapeutic relevance and practical applicability of these systems.

## Figures and Tables

**Table 1:** Composition (%, *w*/*w*) of CEO-loaded microemulsions.

Microemulsion Component	ME-CEO-1	ME-CEO-2	ME-CEO-3
Cinnamon bark essential oil	3.0	3.00	3.0
Isopropyl myristate	1.5	1.50	1.5
Decyl glucoside	30.0	33.75	36.0
Propylene glycol	15.0	11.25	9.0
Aqueous phase *	q.s. ad 100	q.s. ad 100	q.s. ad 100

* The aqueous phase consisted of purified water and a 20% (*w*/*w*) aqueous citric acid solution. The proportion of citric acid solution was adjusted to obtain a final formulation pH of 5.5–6.5.

**Table 2:** Physicochemical properties of the selected O/W CEO-loaded microemulsions.

Code Formulation	Mean Droplet Diameter (nm)	PDI	Dynamic Viscosity (mPa·s)	Refractive Index	Zeta Potential (mV)	Apparent pH
ME-CEO-1	34.62 ± 0.18 ^a^	0.144 ± 0.024 ^a^	26 ± 1.82	1.383 ± 0.003	−0.917 ± 0.12	5.65 ± 0.009 ^a^
ME-CEO-2	31.13 ± 0.51 ^b^	0.312 ± 0.016 ^b^	28 ± 3.27	1.382 ± 0.005	−0.726 ± 0.28	5.59 ± 0.001 ^b^
ME-CEO-3	24.95 ± 0.35 ^c^	0.136 ± 0.023 ^a^	28 ± 1.03	1.381 ± 0.002	−0.927 ± 0.24	5.63 ± 0.004 ^c^

Values are presented as mean ± SD (n = 3). Within each column, different superscript letters (a, b, c) indicate statistically significant differences between formulations, whereas values sharing the same superscript letter are not significantly different (one-way ANOVA followed by Tukey’s post hoc multiple-comparison test, *p* < 0.05). Columns without superscript letters showed no statistically significant differences (*p* > 0.05).

**Table 3:** CEO content and apparent pH of the gelled O/W CEO-loaded microemulsions.

Code Formulation	CEO Content (%)	Apparent pH
MEG-CEO-1	100.78 ± 0.43	6.35 ± 0.006 ^a^
MEG-CEO-2	99.41 ± 0.81	6.27 ± 0.005 ^b^
MEG-CEO-3	100.36 ± 1.15	6.14 ± 0.006 ^c^

Values are presented as mean ± SD (n = 3). Within each column, different superscript letters (a, b, c) indicate statistically significant differences between formulations, whereas values sharing the same superscript letter are not significantly different (one-way ANOVA followed by Tukey’s post hoc multiple-comparison test, *p* < 0.05). Columns without superscript letters showed no statistically significant differences among formulations (*p* > 0.05).

**Table 4:** Apparent viscosity and thixotropy index values of experimental gelled ME formulations.

Formulation Code	Apparent Viscosity (Pa.s)	Thixotropy Index (%)
MEG-1	0.219 ± 0.003 ^a^	46.43
MEG-CEO-1	0.242 ± 0.005 ^a^*	64.72
MEG-2	0.235 ± 0.003 ^b^	46.94
MEG-CEO-2	0.303 ± 0.006 ^b^*	64.87
MEG-3	0.258 ± 0.004 ^c^	49.03
MEG-CEO-3	0.344 ± 0.006 ^c^*	58.74

Values are presented as mean ± SD (n = 3). Statistical analysis was performed by two-way ANOVA, considering DG:PG ratio and CEO incorporation as factors, followed by Tukey’s multiple-comparison test. Different lowercase superscript letters indicate significant differences among DG:PG ratios within the blank or CEO-loaded formulation series, whereas an asterisk (*) indicates a significant difference between each CEO-loaded formulation and its corresponding blank formulation (*p* < 0.05).

**Table 5:** Storage modulus (G’), loss modulus (G”), loss factor (tan δ), and yield stress of the gelled blank and CEO-loaded microemulsions obtained from oscillatory rheological measurements.

Formulation Code	G’(Pa)	G” (Pa)	Loss Factor (tan δ) ^†^	Yield Stress (Pa)
MEG-1	169.62 ± 4.00 ^a^	8.06 ± 4.26 ^a^	0.048	3.450 ± 2.38 ^a^
MEG-CEO-1	206.41 ± 2.77 ^a^ *	13.58 ± 2.84 ^a^	0.065	7.450 ± 1.41 ^a^
MEG-2	193.50 ± 4.80 ^ab^	9.67 ± 0.88 ^a^	0.049	5.001 ± 2.76 ^a^
MEG-CEO-2	385.72 ± 19.13 ^b^ *	16.68 ± 2.06 ^a^	0.043	6.401 ± 3.18 ^a^
MEG-3	213.03 ± 6.42 ^b^	9.01 ± 1.23 ^a^	0.042	5.374 ± 1.62 ^a^
MEG-CEO-3	463.11 ± 7.87 ^c^ *	27.25 ± 5.93 ^b^ *	0.059	9.030 ± 2.55 ^a^

^†^ tan δ = G”/G’. Values are presented as mean ± SD (n = 3). Statistical analysis was performed by two-way ANOVA, considering DG:PG ratio and CEO incorporation as factors, followed by Tukey’s multiple-comparison test. Within each formulation series (blank or CEO-loaded) and each parameter, values sharing at least one superscript lowercase letter are not significantly different, whereas values with no letter in common are significantly different. An asterisk (*) indicates a significant difference between a CEO-loaded formulation and its corresponding blank formulation (*p* < 0.05). For yield stress, two-way ANOVA identified a significant overall effect of CEO incorporation (*p* = 0.02), although individual CEO-loaded versus corresponding blank comparisons did not reach statistical significance after Tukey adjustment.

**Table 6:** Texture parameters determined by the back extrusion test for the blank and CEO-loaded gelled microemulsions.

Formulation Code	Firmness (g)	Consistency (g·s)	Cohesiveness (g)	Index of Viscosity (g·s)
MEG-1	37.02 ± 1.94 ^a^	266.44 ± 5.37 ^a^	−17.76 ± 1.66 ^a^	−82.24 ± 2.71 ^a^
MEG-CEO-1	73.70 ± 5.48 ^a^ *	425.16 ± 8.63 ^a^ *	−39.72 ± 3.51 ^a^ *	−215.81 ± 7.09 ^a^ *
MEG-2	43.56 ± 3.18 ^a^	263.47 ± 6.05 ^a^	−19.33 ± 2.15 ^a^	−96.57 ± 4.38 ^a^
MEG-CEO-2	109.09 ± 7.52 ^b^ *	636.47 ± 8.14 ^b^ *	−64.93 ± 4.27 ^b^ *	−357.17 ± 8.16 ^b^ *
MEG-3	59.52 ± 4.36 ^b^	337.15 ± 4.16 ^b^	−25.50 ± 3.84 ^a^	−144.62 ± 3.59 ^b^
MEG-CEO-3	109.10 ± 6.05 ^b^ *	611.32 ± 6.27 ^c^ *	−64.03 ± 5.92 ^b^ *	−357.81 ± 6.13 ^b^ *

Values are presented as mean ± SD (n = 3). Statistical analysis was performed by two-way ANOVA, considering DG:PG ratio and CEO incorporation as factors, followed by Tukey’s multiple-comparison test. Within each formulation series (blank or CEO-loaded) and each parameter, values sharing the same superscript lowercase letter are not significantly different, whereas values with different letters indicate statistically significant differences among DG:PG ratios. An asterisk (*) indicates a significant difference between a CEO-loaded formulation and its corresponding blank formulation (*p* < 0.05).

**Table 7:** Texture parameters determined by the spreadability test for the blank and CEO-loaded gelled microemulsions.

Formulation Code	Firmness (g)	Work of Shear (g·s)
MEG-1	32.95 ± 1.27 ^a^	25.67 ± 1.43 ^a^
MEG-CEO-1	41.71 ± 1.35 ^a^ *	34.98 ± 1.16 ^a^ *
MEG-2	45.07 ± 2.61 ^b^	36.69 ± 2.14 ^b^
MEG-CEO-2	55.93 ± 2.09 ^b^ *	51.89 ± 2.77 ^b^ *
MEG-3	45.21 ± 1.72 ^b^	38.10 ± 1.55 ^b^
MEG-CEO-3	80.12 ± 2.93 ^c^ *	73.97 ± 2.85 ^c^ *

Values are presented as mean ± SD (n = 3). Statistical analysis was performed by two-way ANOVA, considering DG:PG ratio and CEO incorporation as factors, followed by Tukey’s multiple-comparison test. Within each formulation series (blank or CEO-loaded) and each parameter, values sharing the same superscript lowercase letter are not significantly different, whereas values with different letters indicate statistically significant differences among DG:PG ratios. An asterisk (*) indicates a significant difference between a CEO-loaded formulation and its corresponding blank formulation (*p* < 0.05).

**Table 8:** Physicochemical characteristics of CEO-loaded microemulsions during the 45-day short-term stability assessment.

Formulation Code	Storage Time (Days)	Mean Droplet Diameter (nm)	PDI	Dynamic Viscosity (mPa·s)	Zeta Potential (mV)	Apparent pH
ME-CEO-1	15	34.85 ± 0.21	0.153 ± 0.028	26.1 ± 2.00	−0.912 ± 0.181	5.631 ± 0.124
	30	34.94 ± 0.17	0.150 ± 0.027	25.9 ± 2.06	−0.915 ± 0.147	5.625 ± 0.065
	45	34.75 ± 0.17	0.156 ± 0.020	26.1 ± 2.57	−0.910 ± 0.105	5.638 ± 0.050
ME-CEO-2	15	31.09 ± 0.96	0.318 ± 0.014	28.2 ± 5.01	−0.722 ± 0.333	5.577 ± 0.002
	30	31.27 ± 0.21	0.316 ± 0.013	28.3 ± 4.04	−0.720 ± 0.322	5.570 ± 0.010
	45	31.16 ± 0.65	0.314 ± 0.014	28.1 ± 4.50	−0.725 ± 0.116	5.587 ± 0.015
ME-CEO-3	15	25.04 ± 0.37	0.151 ± 0.032	28.3 ± 1.53	−0.922 ± 0.293	5.618 ± 0.028
	30	24.94 ± 0.36	0.146 ± 0.047	27.8 ± 2.22	0.919 ± 0.265	5.641 ± 0.019
	45	25.03 ± 0.21	0.147 ± 0.039	28.2 ± 1.07	0.924 ± 0.098	5.621 ± 0.011

Values are presented as mean ± SD (n = 5). Day 0 values are reported in Table 2 and were included in the statistical analysis. No statistically significant time-dependent changes were detected for any parameter over the 45-day storage period (*p* > 0.05).

**Table 9:** Physicochemical characteristics of CEO-loaded gelled microemulsions during the 45-day short-term stability assessment.

Formulation Code	Storage Time (Days)	CEO Content (%)	Apparent pH	Apparent Viscosity (Pa.s)	Penetration Value (mm)
MEG-CEO-1	15	100.63 ± 0.57	6.372 ± 0.083	0.249 ± 0.014	573.0 ± 7.0
	30	100.85 ± 0.44	6.344 ± 0.059	0.243 ± 0.014	571.0 ± 17.1
	45	100.70 ± 0.79	6.338 ± 0.042	0.254 ± 0.005	567.7 ± 9.6
MEG-CEO-2	15	99.19 ± 0.99	6.236 ± 0.023	0.307 ± 0.010	538.3 ± 8.6
	30	99.33 ± 0.69	6.255 ± 0.026	0.306 ± 0.024	543.7 ± 11.4
	45	99.26 ± 0.82	6.265 ± 0.013	0.309 ± 0.013	541.3 ± 5.5
MEG-CEO-3	15	100.47 ± 1.77	6.132 ± 0.035	0.348 ± 0.022	438.0 ± 3.0
	30	100.23 ± 1.10	6.154 ± 0.028	0.350 ± 0.019	437.7 ± 4.5
	45	100.33 ± 2.04	6.142 ± 0.010	0.351 ± 0.018	436.7 ± 12.5

Values are presented as mean ± SD (n = 5). Day 0 values are reported in the corresponding initial characterization tables and were included in the statistical analysis. A significant overall effect of storage time was detected only for the apparent pH of MEG-CEO-2 (*p* = 0.035); however, pair comparisons with baseline, adjusted using the Holm procedure, revealed no statistically significant differences (adjusted *p* > 0.05).

**Table 10:** Apparent solubility of CEO in candidate hydroethanolic receptor media evaluated for the in vitro release and permeation studies.

Hydroethanolic Receptor Medium	Apparent Solubility *	Equivalent Apparent Solubility **	Meets Three-Fold Sink Condition (≥0.0041)	Meets Ten-Fold Sink Condition (≥0.0135)
PBS (pH 7.4) + 30% (*v*/*v*) ethanol	30 ± 1.24	0.0033	No	No
PBS (pH 7.4) + 60% (*v*/*v*) ethanol	13.33 ± 0.51	0.0075	Yes	No
PBS (pH 7.4) + 70% (*v*/*v*) ethanol	2.35 ± 0.37	0.0426	Yes	Yes
Normal saline (0.9% NaCl) + 30% (*v*/*v*) ethanol	20 ± 1.46	0.0050	Yes	No
Normal saline (0.9% NaCl) + 60% (*v*/*v*) ethanol	5.71 ± 0.62	0.0175	Yes	Yes
Normal saline (0.9% NaCl) + 70% (*v*/*v*) ethanol	1.6 ± 0.45	0.0625	Yes	Yes

* Volume (mL) of receptor medium required to completely solubilize 0.1 mL of CEO at room temperature. Lower values indicate higher apparent solubilizing capacity. ** Volume (mL) of CEO completely solubilized by 1 mL receptor medium at room temperature.

**Table 11:** Results of kinetic analysis of CEO in vitro release data from gelled microemulsions through a synthetic membrane.

Formulation Code	Zero Order		First Order		Higuchi		Korsmeyer–Peppas		
	K_0_ (µg/h)	R^2^	K_1_ (h^−1^)	R^2^	K_H_ (h^−0.5^)	R^2^	K_P_ (h^−n^)	n	R^2^
MEG-CEO-1	25.543	0.8432	0.6646	0.9676	52.51	0.9819	1.748	0.344	0.9979
MEG-CEO-2	22.831	0.8275	0.4869	0.9567	47.793	0.9738	1.713	0.311	0.9999
MEG-CEO-3	11.106	0.7407	0.2133	0.8916	35.257	0.8882	1.660	0.239	0.9889

**Table 12:** Permeation parameters obtained from the in vitro skin permeation study of the gelled CEO-loaded microemulsions through excised porcine ear skin.

Formulation Code	Jss (µg/cm^2^/h)	K_P_ × 10^−6^ (cm/h)	k_H_ (μg/cm^2^/h^1/2^)
MEG-CEO-1	199.59 ± 3.06 ^b^	66.53 ± 0.95 ^b^	842.22 ± 5.57 ^b^
MEG-CEO-2	214.17 ± 1.13 ^a^	71.39 ± 0.84 ^a^	913.81 ± 6.35 ^a^
MEG-CEO-3	84.70 ± 5.62 ^c^	29.23 ± 1.14 ^c^	514.67 ± 8.27 ^c^

Values are presented as mean ± SD (n = 5). Within each column, different superscript letters (a, b, c) indicate statistically significant differences between formulations, whereas values sharing the same superscript letter are not significantly different (one-way ANOVA followed by Tukey’s post hoc multiple-comparison test, *p* < 0.05).

**Table 13:** Kinetic parameters and coefficients of determination (R^2^) obtained by fitting the in vitro CEO permeation data to different mathematical models.

Formulation Code	Zero Order		First Order		Higuchi		Korsmeyer–Peppas		
	K_0_ (µg/h)	R^2^	K_1_ (h^−1^)	R^2^	K_H_ (h^−0.5^)	R^2^	K_P_ (h^−n^)	n	R^2^
MEG-CEO-1	1.4769	0.6979	0.0238	0.7332	10.9460	0.8724	0.988	0.555	0.9013
MEG-CEO-2	1.3705	0.6507	0.0232	0.6916	11.4830	0.8316	1.081	0.492	0.8783
MEG-CEO-3	1.4778	0.8446	0.0214	0.8763	9.0188	0.9524	0.780	0.664	0.9405

## Data Availability

The authors confirm that the data supporting the findings of this study are available within the article.

## Abbreviations

The following abbreviations are used in this manuscript:

CEO	Cinnamon bark essential oil
APG	Alkyl polyglucoside
CIR	Cosmetic Ingredient Review
IPM	Isopropyl myristate
PG	Propyleneglycol
DEGGE	Diethylene glycol monoethyl ether
DG	Decyl glucoside
Smix	Surfactant/cosurfactant mixture
O/W	Oil-in-water
ME	Microemulsion
ME-CEO	Cinnamon essential oil-loaded microemulsion
PDI	Polydispersity index
DLS	Dynamic light scattering
ELS	Electrophoretic light scattering
LVR	Linear viscoelastic region
SD	Standard deviation
PBS	Phosphate-buffered saline
MEG	Gel-microemulsion
IVRT	In vitro release test
IVPT	In vitro permeation testing
